# Grape Heterogeneity Index: Assessment of Overall Grape Heterogeneity Using an Aggregation of Multiple Indicators

**DOI:** 10.3390/plants12071442

**Published:** 2023-03-24

**Authors:** Claire E. J. Armstrong, Pietro Previtali, Paul K. Boss, Vinay Pagay, Robert G. V. Bramley, David W. Jeffery

**Affiliations:** 1 Australian Research Council Training Centre for Innovative Wine Production, The University of Adelaide, PMB 1, Glen Osmond, SA 5064, Australia; 2 School of Agriculture, Food and Wine, and Waite Research Institute, The University of Adelaide, PMB 1, Glen Osmond, SA 5064, Australia; 3 CSIRO Agriculture and Food, Locked Bag 2, Glen Osmond, SA 5064, Australia

**Keywords:** Cabernet Sauvignon, composite indicator, grape variability, grape maturity, vineyard management, viticulture

## Abstract

Uniform grape maturity can be sought by producers to minimise underripe and/or overripe proportions of fruit and limit any undesirable effects on wine quality. Considering that grape heterogeneity is a multifaceted phenomenon, a composite index summarising overall grape heterogeneity was developed to benefit vineyard management and harvest date decisions. A grape heterogeneity index (GHI) was constructed by aggregating the sum of absolute residuals multiplied by the range of values from measurements of total soluble solids, pH, fresh weight, total tannins, absorbance at 520 nm (red colour), 3-isobutyl-2-methoxypyrazine, and malic acid. Management of grape heterogeneity was also studied, using Cabernet Sauvignon grapes grown under four viticultural regimes (normal/low crop load, full/deficit irrigation) during the 2019/2020 and 2020/2021 seasons. Comparisons of GHI scores showed grape variability decreased throughout ripening in both vintages, then significantly increased at the harvest time point in 2020, but plateaued on sample dates nearing the harvest date in 2021. Irrigation and crop load had no effect on grape heterogeneity by the time of harvest in both vintages. Larger vine yield, leaf area index, and pruning weight significantly increased GHI score early in ripening, but no significant relationship was found at the time of harvest. Differences in the Ravaz index, normalised difference vegetation index, and soil electrical conductivity did not significantly change the GHI score.

## 1. Introduction

Berry physical and chemical heterogeneity is a consequence of grapevine genotype plasticity and the interplay with climate, geophysical parameters, and vineyard management techniques [1,2]. Grape heterogeneity exists at the block, vine, and bunch levels, where each group is nested within the preceding one [3], which is important to consider for sampling regimes, vineyard management, and winemaking purposes. When sorted into maturity classes by density, berries from a single parcel of fruit can have significantly different transcriptome, phenolic composition and extractability, fresh weight (FW), and concentration of sugars, organic acids, anthocyanins, and volatile compounds [4,5,6,7,8,9,10,11]. Grape heterogeneity can therefore be considered a multidimensional phenomenon, although metrics for assessing grape quality for winemaking are often based on simple measurements of sugar (i.e., total soluble solids, TSS), pH, or titratable acidity and rarely consider the distribution of maturity among a grape population.

The variability of berry maturity can be large when grapes initiate ripening, particularly at the bunch-to-bunch level within a vine [3,12]. This leads to differences in the upper and lower limits of the various grape maturity measures that are used, although the differences decrease throughout berry development [5,13]. Similarly, the proportional distribution of berries across maturity classes is more evenly spread at véraison and condenses to maturity classes around the mean as grapes ripen, yielding a smaller standard deviation in grape density as well as grape maturity measures, for example, TSS [7,14,15]. The initiation of berry ripening appears to be related to the ratio of seed weight to berry weight and suggests there is flexibility in hormone regulation of the grape ripening process, although the mechanism of grape maturity synchronisation is not yet understood [15].

High grape heterogeneity translates to a relatively larger proportion of underripe and/or overripe grapes in a parcel of fruit on a single sample date or at the time of harvest [16]. If high grape heterogeneity remains at harvest, there could be significant effects on wine sensory and chemical characteristics [9,14,17]. Therefore, there has been growing interest in managing grape heterogeneity to achieve high-quality wine, with recent work focussing on the use of remote sensing and precision viticulture to address variability across a vineyard [18,19,20]. Differences in vineyard soil type and depth are considered significant driving forces for grape maturity variability as these geophysical parameters correlate to vine vigour and yield, which in turn alter berry composition [20,21].

Conventional viticultural practices involving deficit irrigation and crop load manipulation have been studied for their effects on intra-vine bunch-to-bunch variability [12]. Application of late-season water deficit resulted in berries with higher TSS and anthocyanin content variability, likely due to uneven berry shrivel (dehydration), although crop load had no significant effects. In contrast, other studies showed that crop-thinning led to advanced grape ripening and decreased TSS variability, but the outcome appeared to depend on the mechanism of thinning [22] and, potentially, on the timing of winter pruning [23]. In an alternative approach involving pre-véraison application of 1-naphthaleneacetic acid, an auxin used to delay ripening, the TSS content of treated vines was less variable at the time of harvest than control vines without auxin application [24].

Grape heterogeneity is clearly a complicated and abstruse viticultural issue, not only to control but also to quantify objectively. A single measure of grape maturity variability alone might not succeed in capturing the overall grape heterogeneity of a parcel of fruit and may fail to reveal treatment effects. Results obtained from various measures of grape maturity variability, for example, based on predicted values for berry compositional traits from the absorbance-transmission and fluorescence excitation-emission matrix (A-TEEM) method reported previously [25], could potentially be utilised to make vineyard management decisions, although it would require effort to interpret the results. It is proposed that aggregation of multiple measures of grape maturity variability in the construction of a composite index would provide summarising capabilities and simple comparisons [26]. Indeed, composite indices have been used in viticulture to define aspects such as sustainability [27] and vineyard parameters such as vine health, yield, and geophysical properties [28].

Considering the research gaps, a grape heterogeneity index (GHI) was formulated as an innovative measure of overall grape heterogeneity. The composite index would aid harvest decisions by enabling producers to identify if a parcel of fruit has reached the targeted grape homogeneity and could assist with implementing appropriate vineyard management practices and fruit grading. The approach considered data from measurement of TSS, pH, FW, malic acid, 3-isobutyl-2-methoxypyrazine (IBMP), total tannins, and absorbance at 520 nm (red colour, A520) from Cabernet Sauvignon fruit grown in Coonawarra, South Australia under deficit or full irrigation and normal or low crop load. The GHI was constructed using the sum of absolute residuals of the seven grape maturity measures multiplied by the range of values at the bunch level and applied on multiple dates throughout the 2019/2020 and 2020/2021 vintages to determine the viticultural treatment effects on overall grape heterogeneity. A simplified version of the GHI was also constructed using residuals of TSS, pH, and FW to investigate the relationship between vineyard variability and overall grape heterogeneity.

## 2. Results and Discussion

### 2.1. Exclusion/Inclusion of Grape Maturity Indicators

**Figure 1:**
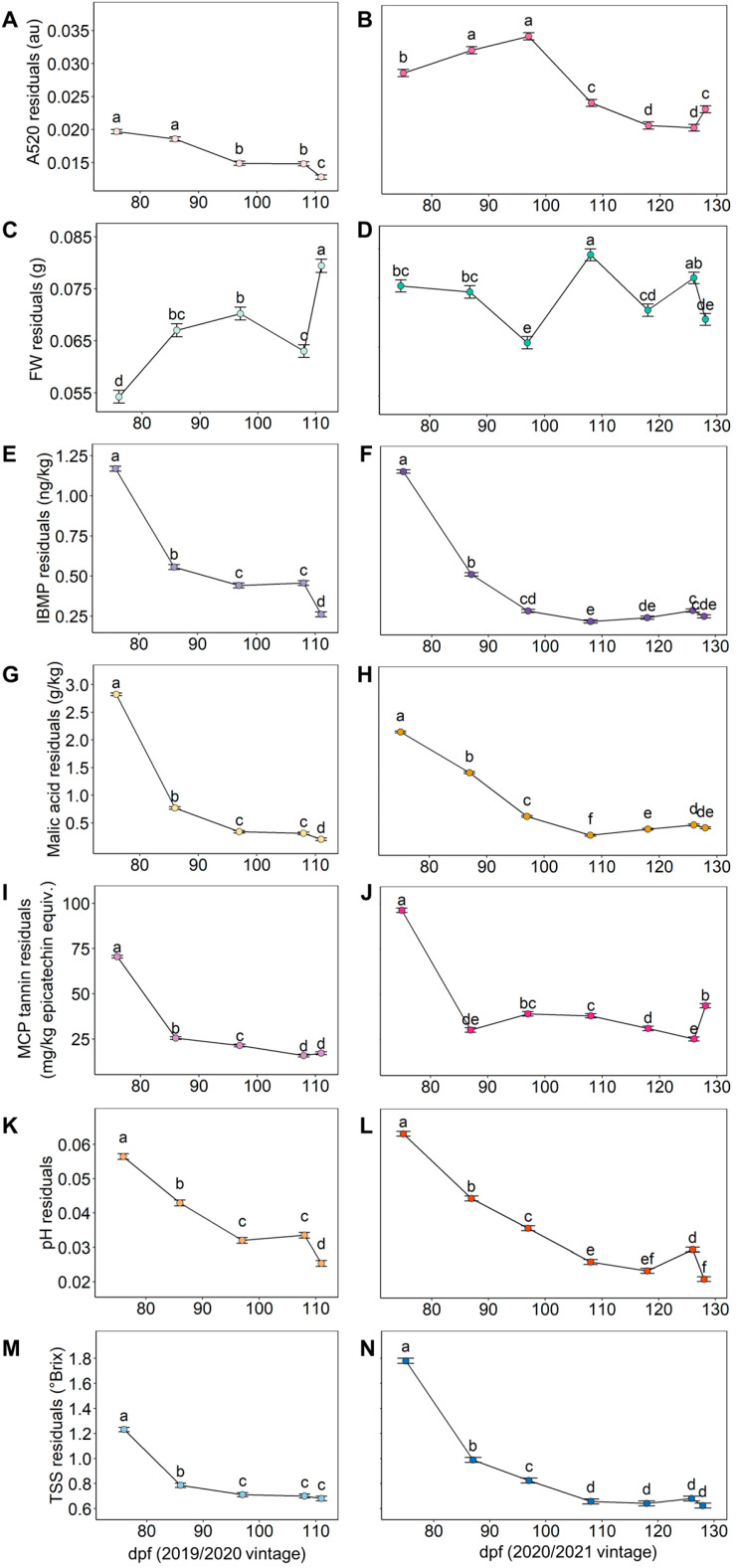
Changes in the bunch-to-bunch variability (absolute residuals) within a vine between sampling dates (days post-flowering, dpf) for (**A**,**B**) absorbance at 520 nm (A520), (**C**,**D**) berry fresh weight (FW), (**E**,**F**) 3-isobutyl-2-methoxypyrazine (IBMP), (**G**,**H**) malic acid, (**I**,**J**) methyl cellulose precipitable (MCP) tannin, (**K**,**L**) pH, and (**M**,**N**) total soluble solids (TSS) over the 2019/2020 (**A**,**C**,**E**, etc.) and 2020/2021 (**B**,**D**,**F**, etc.) seasons according to mean ± SEM (*n* = 24 vines per sampling date). Different lower-case letters within a vintage for a given measurement represent significant differences between sampling date (linear mixed model, α = 0.05, Bonferroni-adjusted).

**Figure 2:**
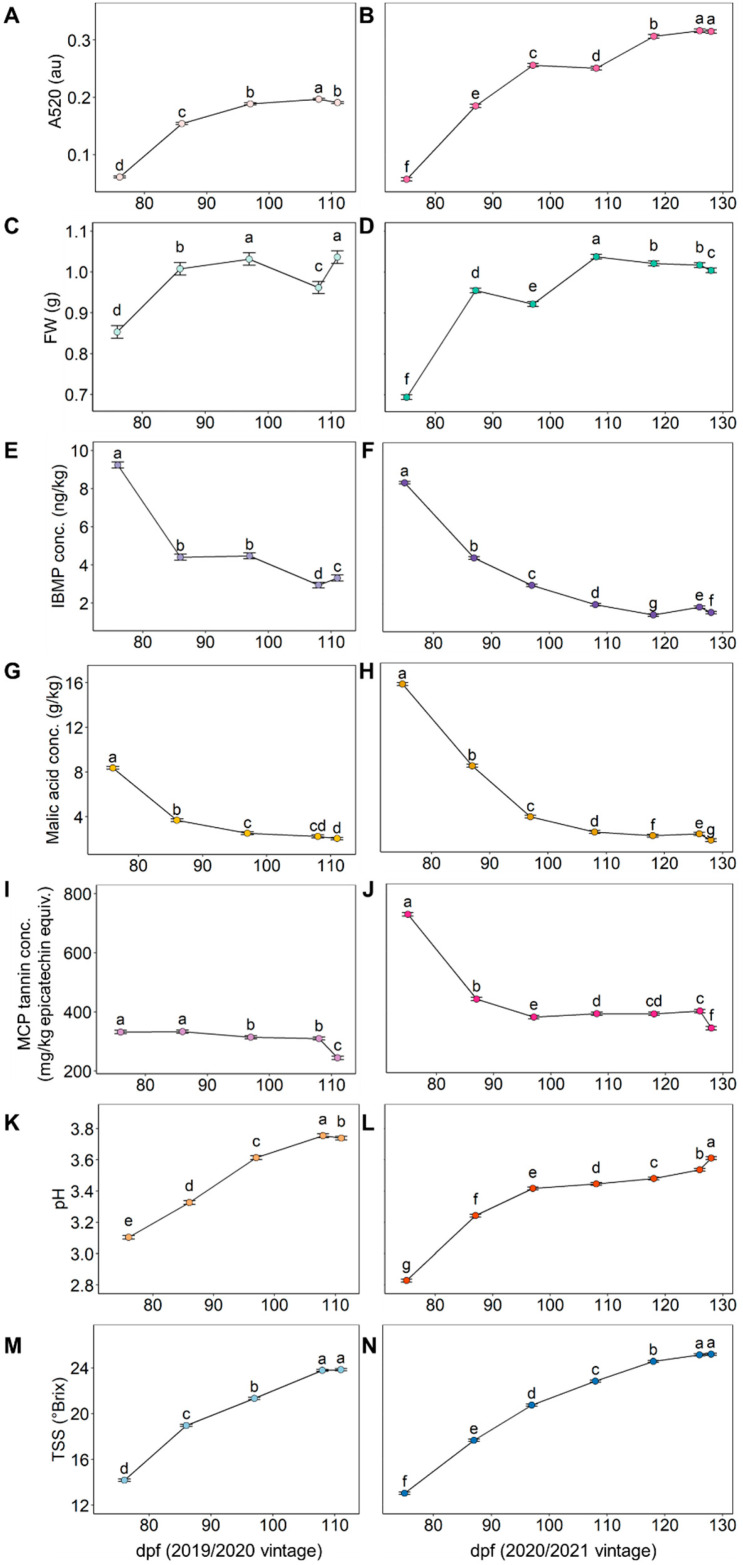
Changes between sampling dates (days post-flowering, dpf) in the average values of (**A**,**B**) absorbance at 520 nm (A520), (**C**,**D**) berry fresh weight (FW), (**E**,**F**) 3-isobutyl-2-methoxypyrazine (IBMP) concentration, (**G**,**H**) malic acid concentration, (**I**,**J**) methyl cellulose precipitable (MCP) tannin concentration, (**K**,**L**) pH, and (**M**,**N**) total soluble solids (TSS) over the 2019/2020 (**A**,**C**,**E**, etc.) and 2020/2021 (**B**,**D**,**F**, etc.) seasons according to mean ± SEM (*n* = 24 vines per sampling date). Different lower-case letters within a vintage for a given measurement represent significant differences between sample date (linear mixed model, α = 0.05, Bonferroni-adjusted).

The applicability of a composite indicator like the GHI originates from the quality of the input data; therefore, the relevance to winemaking and the accuracy of several grape maturity indicators as measures of technological, phenolic, and flavour maturity that can be predicted with a rapid A-TEEM technique [25] were thoroughly evaluated as a first step. Grapes were sampled multiple times throughout the ripening period, and differences in bunch-to-bunch absolute residuals extracted from the LMMs (Figure 1) and average values (Figure 2) of grape maturity indicators were analysed. Due to the hierarchal structure of the dataset, there was a requirement to account for non-independence between data points. Therefore, the use of LMMs with nested random effects (bunch, vine, and block) provided increased model power and minimised false-positive associations by correcting for this specific data structure. Among the scales of possible heterogeneity involving vineyard, vine, and bunch, intra-vine bunch variability was shown to be the most significant source of variation in berry composition and FW throughout ripening (Table S1 of the Supporting Information), hence the intra-vine bunch variability was considered herein. This finding aligned with a previous report [12], and although intra-bunch variability was not examined in the current study, it was still accounted for in LMMs because it can potentially contribute considerably to variability [3]. Vintages were analysed separately due to significant differences (*p* ≤ 0.05) in average values and residuals of grape maturity measures between the two growing seasons (Figures S1 and S2 of the Supporting Information).

Figure 1 shows that pH residuals in both vintages and A520, IBMP, malic acid, and MCP tannin residuals in the 2019/2020 vintage decreased from the initial sampling date until harvest. Fresh weight residuals in 2019/2020 significantly increased from 76 dpf to 97 dpf and then decreased by 108 dpf before significantly increasing to the highest FW residual value of the vintage. Residuals in 2020/2021 for A520, FW, and MCP tannin fluctuated throughout the sampling period, and IBMP and malic acid residuals initially decreased, but as fruit matured, there was an increase in bunch-to-bunch variability. For TSS residuals in both vintages, there was an initial decline, and then residuals remained constant from 97 to 111 dpf in 2019/2020 and 108 to 128 dpf in 2020/2021.

Previous studies that analysed the variability of TSS, pH, tannins, grape colour, FW, and malic acid, reported similar trends to the current study [4,5,13], although the mechanisms that account for the observations are uncertain. For FW variability, uneven berry cell death within a bunch could be a main driving force as well as the irrigation regime [1]. For berry chemical parameters, it is suggested that environmental factors [24,29,30,31] and geophysical characteristics of a vineyard [18,20] are responsible for the trends in grape heterogeneity over time.

The analysis of IBMP variability at bunch and vine levels has seemingly not been reported before but has been shown to be a dynamic feature of a vineyard at the block level [32]. Furthermore, vine vigour differences were deemed to result in spatial variability in IBMP across a vineyard [21].

Average values of TSS, pH, IBMP, malic acid, absorbance at 520 nm, FW, and MCP tannin were determined for multiple dates in the 2019/2020 and 2020/2021 vintages (Figure 2). The changes in A520, pH, and TSS were inverse to their residual counterparts; as averages increased, variability decreased. For malic acid, IBMP, and MCP tannin, residuals decreased as ripening increased. Interestingly, the average values and residuals of FW appeared to follow a similar trend to each other (Figure 1 and Figure 2). Despite an early harvest in the 2020 vintage, TSS values still reached 23.8 °Brix at 111 dpf, although they were higher at harvest in 2021, at 25.2 °Brix (Figure 2).

**Figure 3:**
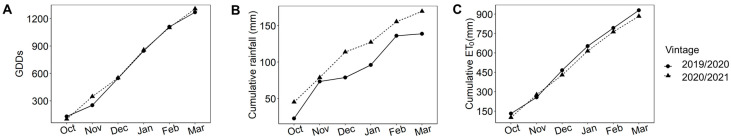
Cumulative growing season data showing (**A**) growing degree days (GDDs), (**B**) rainfall, and (**C**) evapotranspiration (ET_0_) during the 2019/2020 and 2020/2021 vintages.

The 2019/2020 growing season (i.e., October to March) was characterised by five frost events throughout October and a late frost in November 2019 around flowering, followed by low rainfall and two heatwaves (defined as three or more days with maximum temperature above 35 °C) in December and one in January. In comparison, the 2020/2021 growing season had one frost early in October and a cooler December and January, with one heatwave in January. However, cumulative GDDs over the 2019/2020 and 2020/2021 growing seasons appear to be comparable (Figure 3A). The cumulative rainfall was slightly different between the two growing seasons as 2019/2020 had 18% less rainfall over the six-month period than 2020/2021 (Figure 3B), but cumulative ET_0_ was comparable between the two vintages (Figure 3C). Therefore, it could be concluded that the timing of weather events plays a role in increasing or decreasing grape heterogeneity [1,23,33].

This assessment of grape maturity measures that cover different quantitative indices of maturity [25] demonstrated their relevance to the definition of overall grape heterogeneity. Although examined, CIRWG and tartaric acid were excluded from the construction of the GHI. It appeared that CIRWG, an index correlated to anthocyanin concentration and composition [34], was unable to describe the expected trend in grape colour. Samples at 75 dpf in the 2020/2021 vintage had the highest CIRWG value (Figure S3 of the Supporting Information), potentially due to grape seeds being incorporated in the homogenates used in this study that have been shown to have higher colourimetric values than grape skins and higher colourimetric values before seed browning occurs later in ripening [34,35]. However, considering that grape colour is an important contributing factor to red wine quality and increases due to the biosynthesis of anthocyanins, A520 was used as an indicator for grape colour instead [36]. In general, tartaric acid residuals were lower on later sampling dates (Figure S4 of the Supporting Information), which is similar to results previously reported [5]. Tartaric acid could possibly be used but was excluded from GHI construction because of missing values for multiple sample dates. Finally, with IBMP typically being the dominant methoxypyrazine, it was decided not to include IPMP and SBMP in the GHI construction as the concentrations were below the limits of detection, being 0.11 and 0.15 ng/kg, respectively, on later sampling dates.

### 2.2. Viticultural Regime Effects on Individual Indicator Variability

**Figure 4:**
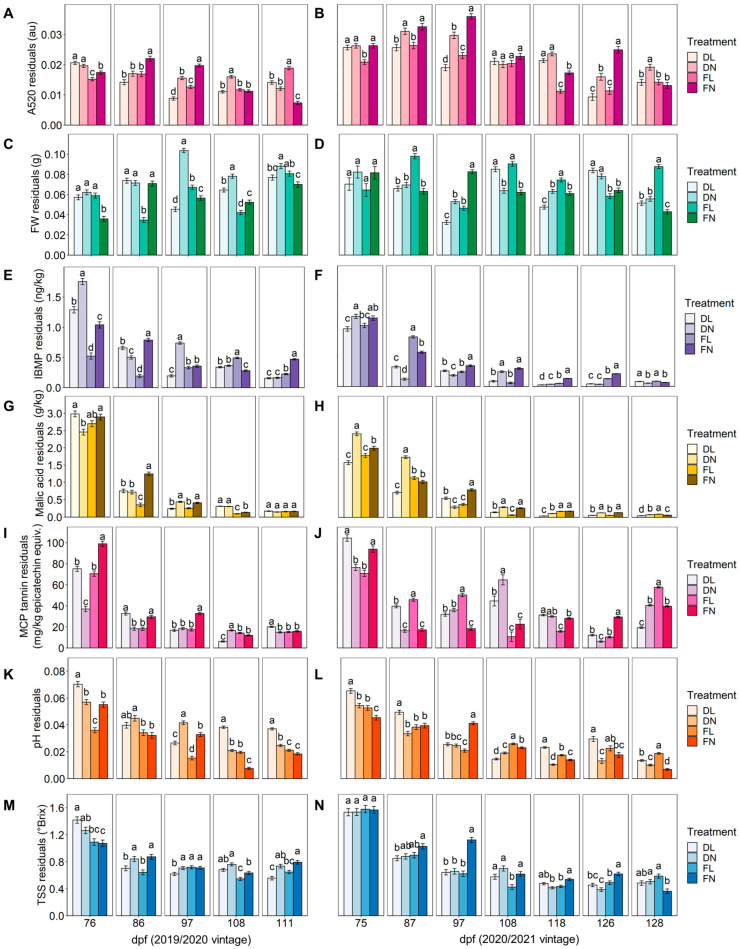
Bar charts showing the changes in the bunch-to-bunch variability (absolute residuals) in response to crop load and irrigation regimes for (**A**,**B**) absorbance at 520 nm (A520), (**C**,**D**) berry fresh weight (FW), (**E**,**F**) 3-isobutyl-2-methoxypyrazine (IBMP), (**G**,**H**) malic acid, (**I**,**J**) methyl cellulose precipitable (MCP) tannin, (**K**,**L**) pH, and (**M**,**N**) total soluble solids (TSS) for each sampling date (days post-flowering, dpf) in the 2019/2020 (**A**,**C**,**E**, etc.) and 2020/2021 (**B**,**D**,**F**, etc.) seasons. Bars and error bars represent the mean ± SEM (*n* = 6 vines per treatment). Different lower-case letters on a given sampling date represent significant differences between treatments (linear mixed model, α = 0.05, Bonferroni-adjusted). DL = deficit irrigation/low crop load, DN = deficit irrigation/normal crop load, FL = full irrigation/low crop load, FN = full irrigation/normal crop load (grower control).

Scrutinising the underlying indicators of grape maturity variability that are to be incorporated in the proposed GHI allows for evaluation of the potential ability of the composite indicator to determine viticultural treatment impacts on grape heterogeneity. Therefore, viticultural treatment effects on the residuals from the determination of A520, FW, IBMP, malic acid, MCP tannin, pH, and TSS were compared on individual sampling dates in 2019/2020 and 2020/2021 (Figure 4). Interestingly, at the time of harvest, FN (grower control) variability was significantly lower than FL, DN, and DL treatments for only two out of seven measures (A520 and FW) in 2019/2020 and significantly lower in IBMP, TSS, and pH residuals in 2020/2021. On the other hand, FN treatment had higher TSS and IBMP residuals in 2019/2020, and treatment FL had higher A520 variability in the 2019/2020 vintage but significantly higher FW, malic acid, MCP tannin, pH, and TSS residuals in 2020/2021. For treatment DL in the 2019/2020 vintage, TSS and IBMP residuals were significantly lower at the time of harvest, and pH and MCP tannin residuals were higher, whereas in 2020/2021, malic acid and MCP tannin residuals were significantly lower. Treatment DN consistently had neither significantly lower nor higher variability in all measures in both vintages, except for FW residuals being significantly higher in 2019/2020. Treatment effects on grape maturity variability measures fluctuated on other sampling dates in both vintages.

Variations in yield per vine and Ravaz index (i.e., kg vine yield/kg pruning weight) can influence grape maturity, and significant differences in these parameters existed between normal and low crop load treatments in the 2020/2021 vintage but not in 2019/2020, although low crop load vines appeared to be 30% lower in yield (Table S2 of the Supporting Information). Understandably, the bunch count was significantly lower for low-cropped vines in both vintages. Results suggested that the chosen crop load adjustment was such that the FL treatment was not source limited and reached a higher TSS concentration than the FN treatment in the 2020/2021 vintage [12,33], but the viticultural treatments imposed in the current study seemed not to have delayed overall ripening consistently over two vintages (Figure S5 of the Supporting Information). Differences in vine yield between vintages were significant (*p* ≤ 0.001, Table S2), most likely due to the late frost in the 2019/2020 season affecting the fruit set, thereby altering the source-sink ratio and changing crop load treatment effects. Indeed, changing the source-sink ratio of vines can also cause a delay in ripening. The photosynthetic assimilation rate for deficit irrigation vines was significantly lower on multiple sampling dates (Table S3 of the Supporting Information), possibly due to the water stress these vines experienced earlier in both vintages, which may have caused the vines to be source limited compared to fully irrigated vines. However, there appeared to be an increase in grape TSS concentration at the time of harvest in deficit irrigation treatments, possibly due to berry dehydration, although deficit irrigation did not consistently increase grape heterogeneity across multiple measures in both vintages (Figure 4). Pruning weights from the viticultural treatments were comparable in the 2020/2021 vintage, and LAI was not significantly different in 2020/2021 but was significantly higher for fully irrigated vines and low-cropped vines in the 2019/2020 vintage (Table S2).

Crop load manipulations explored by Calderon-Orellana et al. [12] in California to manage Cabernet Sauvignon grape heterogeneity at harvest were comparable to the 2020/2021 vintage crop load of the current study. That previous work showed that green-drop and upper bunch thinning had no significant effect on grape heterogeneity, which may have been due to both crop load treatments not being source limited. Those researchers also found that late season water deficit increased grape maturity variability, suggesting that the timing (early or late season) of water deficit is important for grape heterogeneity [1].

Overall, crop load and irrigation treatments appeared to have significant main and interaction effects on the variability of grape maturity measures on each sampling date (Table S4 of the Supporting Information) but the effects were inconsistent (Figure 4). Numerous environmental parameters appear to contribute to the variability of grape maturity measures and may play a role in the inconsistent outcomes from viticultural treatments. Furthermore, there were differences in soil EC_a_ across the Treatment Block (Figure S10), which has been shown to contribute to vineyard-scale grape heterogeneity [18,20]. Notably, treatment effects on overall grape heterogeneity were difficult to determine by assessing the individual data sets (maturity measures) presented in Figure 4, thereby highlighting the need for a composite indicator such as the proposed GHI for research and industry use.

### 2.3. Calculation, Normalisation, Aggregation, and Weighting Methods

**Figure 5:**
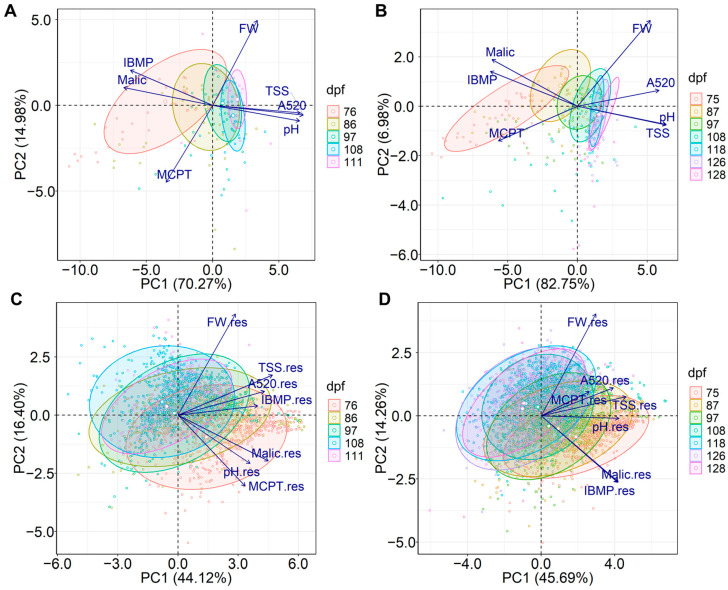
Biplots showing the first two principal components (PC1 and PC2) for PCA of (**A**,**B**) average values and (**C**,**D**) log of absolute residuals (res) for absorbance at 520 nm (A520), berry fresh weight (FW), 3-isobutyl-2-methoxypyrazine (IBMP), malic acid (malic), methyl cellulose precipitable (MCP) tannin, pH, and total soluble solids (TSS) grouped by sampling date (days-post-flowering, dpf) in 2019/2020 (**A**,**C**) and 2020/2021 (**B**,**D**).

PCA was performed on bunch averages and absolute residuals for the seven grape maturity indices to assess the interrelationships between individual indicators and guide decisions regarding GHI calculation, normalisation, and aggregation methodology, and finally, to gain insight into the underlying structure of the GHI. The PCA biplots in Figure 5 show the first two principal components, PC1 and PC2, with scores grouped by sample date (dpf). The cumulative variance explained by PC1 and PC2 in the 2020/2019 vintage was 85.3% and 60.5% for average maturity index values and residuals, respectively (Figure 5A,C) and that for 2020/2021 was 89.7% and 59.9%, respectively (Figure 5B,D).

For the grape measurement average values, the separation of samples along PC1 was similar in both vintages (Figure 5A,B) and related to differences in grape maturity. Later sampling dates moved to the right of PC1, which was positively correlated to values for TSS, red colour (A520), pH, and less so to FW, and negatively correlated to malic acid, IBMP, and MCP tannins. Separation of samples along PC2 is driven primarily by differences in FW and MCP tannin being opposite in the 2019/2020 vintage and mostly FW in the 2020/2021 vintage, suggesting that these variables are less dependent on grape maturity. Encouragingly, these results highlighted that the seven maturity indices used in this study sufficiently capture differences in grape samples. Of note, the confidence ellipses are larger for earlier sample dates in both vintages and decrease for later sample dates (Figure 5A,B). As such, range could be used as a measure of variability to differentiate fruit parcels and was therefore considered when formulating the GHI (Equation (1) in Section 3.8.6). Yet, from 108 dpf in the 2019/2020 vintage and 118 dpf in the 2020/2021 vintage, there appeared to be minimal change in the sample date PC1 score range, indicating a plateau in variability as determined by range.

In both vintages, all seven measures of grape maturity variability, i.e., absolute residuals of TSS, FW, IBMP, malic acid, MCP tannin, A520, and pH, were positively correlated to PC1 (Figure 5C,D), revealing that the separation of samples was again related to differences in grape maturity. Consideration of the separation by sample date along PC2 suggests there is an interaction effect among the aspects of grape maturity. Separation according to maturity along PC2 appears to be primarily due to differences in FW and MCP tannin in the 2019/2020 vintage, with the two variables having a low correlation to each other (r = 0.37). In the 2020/2021 vintage, samples are moved in the positive direction along PC2 by higher FW residuals and lower malic acid and IBMP residuals. FW residuals had a weak relationship with malic acid (r = 0.32) and IBMP (r = 0.33), but residuals of the latter two variables were highly correlated (r = 0.78). This suggests that factors driving FW variability do not necessarily contribute to grape chemical variability as expressed by MCP tannin, malic acid, or IBMP residuals. Altogether, the biplots with residuals shown in Figure 5C,D describe the complex phenomenon of grape heterogeneity as the fruit matures, depicting the lessening in grape maturity variability throughout the ripening process but also demonstrating the remaining grape heterogeneity in the later ripening stages.

The positive correlations to PC1 of the residuals of seven grape maturity indices (as indicators of grape heterogeneity) suggested an additive aggregation of residuals would be appropriate to formulate the GHI, thus aligning with the objective to define the overall grape heterogeneity of a given parcel of fruit. Nonetheless, the use of PC loadings rather than absolute residuals could also be considered for further composite index construction. In the present case, the min-max scaling of individual heterogeneity indicators was deemed appropriate so that values were on the same scale between 0 and 1. Log transformation was applied before PCA because maturity measure residuals were positively skewed. Such a transformation step would also need to be considered when formulating the GHI.

Importantly, the contributions of each measure of grape maturity variability to PC1 (Figure 5C,D) were not equal, implying that different weights should be applied to individual indicators. Despite this, equivalent weighting was considered due to the relatively equal importance of the indicators from a winemaking perspective, especially in terms of the significance of IBMP to Cabernet Sauvignon as used in this work. The main drawback of such an approach could be the risk of “double counting” IBMP and malic acid residuals, considering these indicators were highly correlated based on the 2020/2021 vintage data (Figure 5D). Nevertheless, the association of these two measures might not indicate a shared latent phenomenon, so both measures were included in the formulation of the GHI (according to Equation (1) in Section 3.8.6). Equal fractional weights for each maturity index residual were thus applied, giving GHI scores ranging between 0 and 1 that could be interpreted as percentages if necessary.

### 2.4. Uncertainty and Sensitivity Analysis

**Figure 6:**
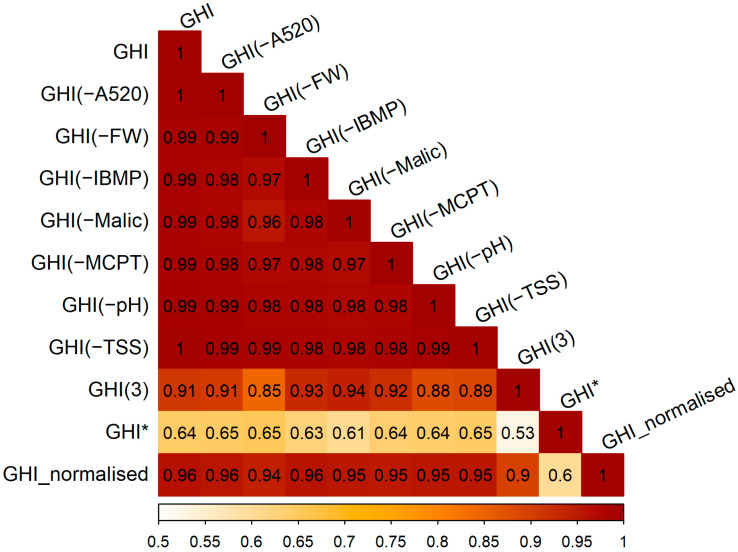
Correlation plot used to assess uncertainty with a multimodelling approach, showing the relationship between GHI calculated using Equation (1) with seven variability indicators, and GHI with six indicators with the singular exclusion of absorbance at 520 nm (GHI(−A520)), berry fresh weight (GHI(−FW)), IBMP (GHI(−IBMP)), etc. along with GHI with TSS, FW, and pH as indicators (GHI(3)), an alternate GHI described in Equation (2) (GHI*), and GHI calculated with Equation (1) from residuals that were square root transformed and normalised (GHI_normalised). Darker red squares indicate a higher Pearson correlation coefficient.

With the approach to calculation, aggregation, and normalisation determined, uncertainty and sensitivity analyses were performed to assess the robustness of the GHI, as suggested when constructing a composite index [26]. Potential sources of error were deemed to be the selection of grape variability indicators and the calculation and normalisation methods. Therefore, the inclusion/exclusion of individual indicators (e.g., GHI (−TSS)), use of a different formula (Equation (2) in Section 3.8.6, GHI*), and changing the transformation and normalisation steps (GHI_normalised) were correlated with GHI (Figure 6).

The Pearson correlation coefficients (r) were between 1.00 and 0.53, indicating very low to moderate uncertainty of the GHI. The major source of uncertainty was determined to be the calculation step, based on the value of r being lower for GHI* than GHI_normalised or in the cases where an indicator was excluded (Figure 6). Interestingly, a GHI developed with only TSS, FW, and pH (i.e., GHI(3)), of which TSS and pH are longstanding technological maturity indicators, had a strong correlation (r = 0.91) with the more complex GHI, which included all seven indicators (incorporating information on maturity with regard to phenolic and flavour compounds along with technological maturity [25]).

**Figure 7:**
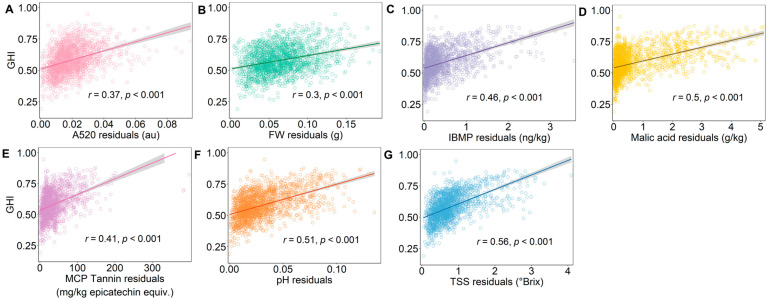
Sensitivity analysis achieved using scatterplots showing the relationship between bunch-to-bunch (i.e., within a vine) GHI score and residuals from linear-mixed models for (**A**) absorbance at 520 nm (A520), (**B**) berry fresh weight (FW), (**C**) 3-isobutyl-2-methoxypyrazine (IBMP), (**D**) malic acid, (**E**) methyl cellulose precipitable (MCP) tannin, (**F**) pH, and (**G**) total soluble solids (TSS). Linear fits (–) are graphed to aid visualisation of the relationship between variables, with Pearson correlation coefficient (r) and *p*-value indicating the strength and significance of the relationship.

Scatterplots between the input (grape maturity measure residuals) and output (GHI scores calculated with Equation (1)) show positive and moderate to strong correlations (Figure 7A–G) with significant relationships between variables (*p* ≤ 0.001), suggesting the GHI score relates to changes in the individual grape maturity indices. Overall, the robustness of the GHI appears to be suitable, but ultimately, its applicability to summarising the overall grape heterogeneity of a given fruit parcel required final testing.

### 2.5. Viticultural Regime Effects on Overall Grape Heterogeneity

**Figure 8:**
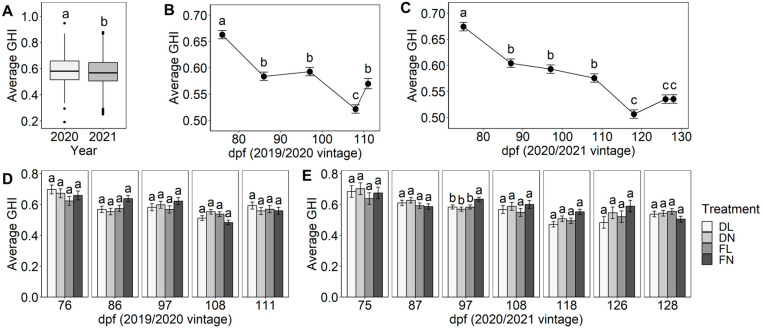
Changes in the bunch-to-bunch variability determined by GHI score according to (**A**) vintage and sampling date (dpf) per season for (**B**) 2019/2020 and (**C**) 2020/2021, and in response to crop load and irrigation regime (*n* = 6 vines per treatment) for each sample date in (**D**) 2019/2020, and (**E**) 2020/2021. Bars/points and associated error bars represent the mean ± SEM of GHI score. Different lower-case letters for a given year or sampling date represent significant differences between sampling dates or treatments (linear mixed model, α = 0.05, Bonferroni-adjusted). DL = deficit irrigation/low crop load, DN = deficit irrigation/normal crop load, FL = full irrigation/low crop load, FN = full irrigation/normal crop load (grower control).

The usefulness of the proposed GHI for summarising overall grape heterogeneity between vintages (Figure 8A), sample dates (Figure 8B,C), and viticultural regimes (Figure 8D,E) was investigated. There was a significant difference in average GHI score between the 2019/2020 and 2020/2021 seasons, albeit with 2019/2020 having a higher GHI score by only 0.03. In general, the GHI score demonstrated similar trends in both seasons of the study, with scores decreasing from the first sample date to harvest, as could be expected. In the 2019/2020 vintage, the GHI score decreased from an average of 0.66 at 76 dpf to 0.52 at 108 dpf but then significantly increased within three days to 0.57 at 111 dpf. From individual indicator analysis (Figure 1C), it was shown that fruit of greater maturity had higher FW residuals, which may be driving the increase observed for GHI score at 111 dpf in the 2019/2020 vintage. There was a significant decrease in GHI score from 0.67 at 75 dpf to 0.51 at 118 dpf in the 2020/2021 vintage, but interestingly, no significant change in overall grape heterogeneity from 118 dpf through to harvest at 128 dpf occurred (GHI score between 0.51–0.54). From Figure 1, it appears that the combination of TSS residuals remaining constant and the fluctuations in FW, A520, IBMP, malic acid, MCP tannin, and pH residuals resulted in a constant GHI score for the last three sampling dates. Therefore, it could be suggested that fruit should be harvested once GHI scores plateau and other grape maturity targets are met. Furthermore, grape heterogeneity targets could be set with grading from “very high” to “very low” based on GHI scores to aid interpretation by industry practitioners. The proposed GHI presents a novel starting point, but additional research into the effects of any remaining grape heterogeneity at harvest is required to understand the optimal GHI score for desirable wine chemical and sensory properties (perhaps on a varietal basis).

Deficit/full irrigation and normal/low crop load treatment effects were not significantly different when grapes were less mature (76 and 75 dpf) in both seasons (Figure 8D,E) and were not significant by the time of harvest at 111 and 128 dpf. This result summarises the fluctuations in TSS, red colour, pH, IBMP, malic acid, MCP tannin, and FW residuals observed at harvest for treatments (Figure 1), showing that the GHI score respects the general trends of the underlying individual variability indicators.

In summary, GHI ranking appeared to be a valuable tool to determine bunch-to-bunch grape heterogeneity of a parcel of fruit, although admittedly, numerous measurements are required (noting that the simplest version GHI(3) also performed relatively well; Section 2.4). Undertaking those analyses can involve time-consuming and expensive techniques that limit the accessibility, speed, and ease of implementation of the GHI. Fortunately, a rapid spectroscopic approach has been recently developed for predicting multiple grape maturity indices from one analytical approach [25], which could greatly improve the availability of data for vineyard managers and decrease the costs involved with constructing and implementing a GHI.

### 2.6. GHI Relationship with Vineyard Variability

Vine canopy architecture parameters (leaf area index, LAI and normalised difference vegetation index, NDVI), vine size (vegetative growth and yield), and soil EC_a_ have been shown to correlate to grape maturity measures [19,20,21,37], and Figure 1 and Figure 2 demonstrate the change in grape heterogeneity relative to maturity. As such, this investigation considered whether overall grape heterogeneity might have an inverse relationship with these vine attributes and soil EC_a_. Grape samples were collected from 30 vines within the Commercial Block at 75, 108, and 128 dpf in the 2020/2021 vintage. The residuals of the easily measurable parameters of TSS, FW, and pH were processed at the vine level due to vineyard spatial variability primarily affecting vine-to-vine attributes, and the simplest calculation of GHI(3) was implemented. As shown earlier in Figure 6, GHI(3) had a strong correlation to the more complex GHI with seven aggregated variability indicators and was therefore deemed suitable for an initial investigation into the relationship of overall grape heterogeneity with soil EC_a_ and vine attributes.

**Figure 9:**
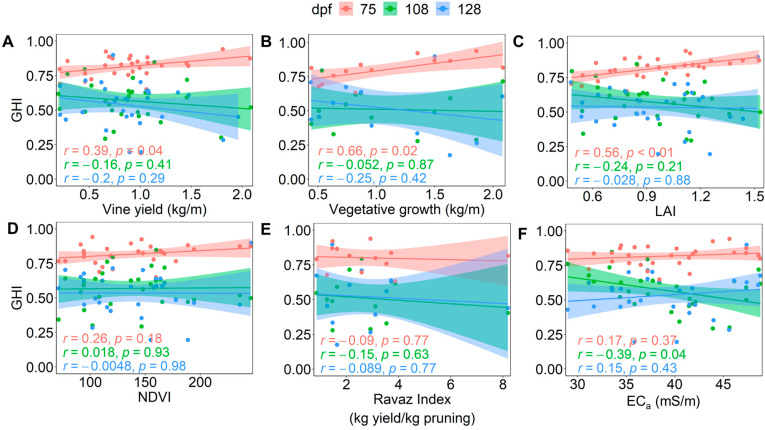
Relationship of vine GHI(3) scores with scaled (**A**) vine yield, (**B**) vegetative growth, (**C**) leaf area index (LAI), (**D**) normalised difference vegetation index (NDVI), (**E**) Ravaz index, and (**F**) soil electrical conductivity (EC_a_) across the Commercial Block for different sampling dates (75, 108 and 128 dpf) in season 2020/2021. Linear fits (–) are graphed to aid visualisation of the relationship between variables, with Pearson correlation coefficient (r) and *p*-value indicating the strength and significance of the relationship.

Vine GHI(3) scores were shown to have a significant relationship with yield, vegetative growth, and LAI at 75 dpf (Figure 9A–C), revealing that larger vine canopies and yields had higher overall grape heterogeneity early in ripening. The strength of the correlations was low to moderate, however, with r = 0.39–0.66 for GHI(3) score versus vine yield, vegetative growth, and LAI. Evaluation of Figures S6–S11 (Supporting Information) reveals that vines with a higher yield, pruning weight, and LAI had significantly lower TSS values and significantly higher TSS variability at 75 dpf. Furthermore, vines with higher LAI had significantly lower pH values and higher pH residuals, and vines with higher LAI and/or pruning weight had significantly lower berry FW values and higher FW residuals at 75 dpf. This indicated that the vines with larger canopies and yields had less mature fruit on this sampling date, which, in turn, would be expected to have higher variability, as observed in Figure 9A–C. Interestingly, by 108 dpf there is no longer a significant relationship between LAI, vegetative growth, and yield with GHI(3) score.

There were inconsistent and insignificant relationships for GHI(3) score with vine crop load, NDVI, and soil EC_a_ on all three sample dates (Figure 9D–F), except for the significant relationship (*p* = 0.04) with EC_a_ at 108 dpf (Figure 9F), when a lower GHI(3) score seemed to depend on soil with higher EC_a_. Interestingly, there were no significant relationships between berry maturity indices or their residuals and the Ravaz index (Figure S11), despite this parameter being considered an important aspect of grape maturity and wine quality [37]. Assessment of the residuals of individual indicators (Figures S6–S11) suggested that a change in vine canopy architecture, soil EC_a_, yield, or crop load (Ravaz index) may change average berry FW, TSS, or pH, but this does not always result in a significant change in variability. This accords with previous research, which concluded that grape heterogeneity did not correlate with pruning weight, vine yield, or crop load [12]. As a final remark, the GHI(3) score appears to sufficiently describe the relationship, or lack thereof, between overall grape heterogeneity and vine attributes and soil EC_a_ on multiple sampling dates, although further research could consider other grape chemical parameters.

## 3. Materials and Methods

### 3.1. Climate Observations

Monthly mean temperature, average monthly rainfall, and base evapotranspiration (ET_0_) values for 2019/2020 and 2020/2021 were obtained from the Bureau of Meteorology’s Coonawarra weather station 026091 in South Australia at 37.29° S, 140.83° E. Comparisons of the 2019/2020 and 2020/2021 growing seasons were undertaken using cumulative growing degree days (GDDs) on a monthly basis (October to March) with a base temperature of 10 °C [38], cumulative rainfall, and ET_0_.

### 3.2. Vineyard Site

**Figure 10:**
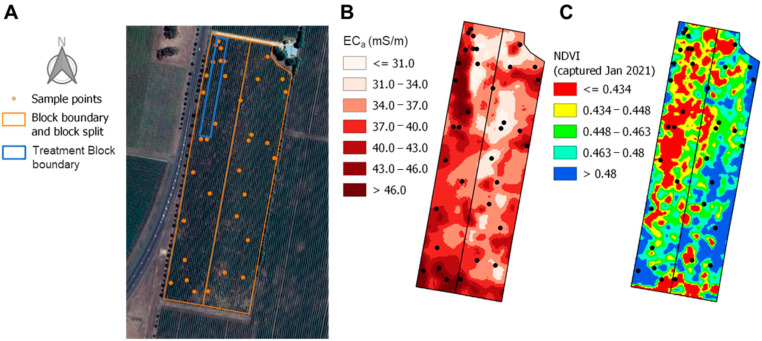
Commercial Block in Coonawarra (background image Copyright (2022), Google) comprised of vines planted in 1976 (west-side) and 1996 (east-side) showing (**A**) sampled vines (orange dots), block boundary with blocks split by vine age (outlined with orange boxes), and Treatment Block (blue box), (**B**) Variation in soil electrical conductivity (EC_a_, mS/m) surveyed by EM38 in 2011, and (**C**) Normalised difference vegetation index (NDVI) acquired at 63 dpf (January 2021) at 80 cm resolution prior to scaling. Images generated using QGIS version 3.18.0.

*Vitis vinifera* L. cv. Cabernet Sauvignon vines (Reynella clone) grown in a 7.32 ha commercial vineyard (Commercial Block, Figure 10) in Coonawarra, South Australia (37.38° S 140.84° E, 57 m above sea level) were sampled during the 2019/2020 and 2020/2021 seasons. The Commercial Block was situated on Terra Rossa soils: gradational clay loam over calcrete with various shallow soils, including silt and coarse and fine sand [39]. Vines were planted in 1976 (west block) and 1996 (east block), with north/south row orientation and 1.83 m × 3.38 m spacing (vine × row). All vines were planted on their own roots, vertically trained with sprawling canopy, and cordons were mechanically spur-pruned with hand-pruning adjustments to two nodes per spur after machine pruning. Spur density was estimated in the 2019/2020 vintage to be 18 ± 0.1 spurs/m of cordon. Eutypa was present in the vineyard (approximately 60% of vines were affected), and effort was made to select vines without disease according to visual examination. Vines were frost irrigated prior to frost events to a total of 0.69 ML/ha in 2019/2020 and 0.13 ML/ha in 2020/2021, and drip-irrigated from November to March to a total of 1.19 ML/ha in 2019/2020 and 0.89 ML/ha in 2020/2021.

### 3.3. Viticultural Treatments

Treatments were applied as detailed previously [25]. In summary, triplicate treatments were set up as a 2 × 2 factorial split block design within a 0.6 ha area (Treatment Block, Figure 10A) of the Commercial Block. Sustained deficit irrigation was implemented 21 days post flowering (dpf) in 2019 within the southern half of the Treatment Block. Deficit irrigation vines received approximately 40% of crop evapotranspiration (ET_c_) compared to full irrigation vines, which received approximately 60% of ET_c_. The treatments received the same frost irrigation, but deficit irrigation vines received approximately 50% less drip irrigation through the growing season than full irrigation. The crop coefficient (k_c_) values were estimated using a recent study in Coonawarra [40]. The northern half of the Treatment Block remained at full irrigation (control). Within each irrigation system, two crop load treatments were established at 56 dpf and 58 dpf in 2020 and 2021, respectively. The low crop load treatment vines underwent removal of every distal bunch on a cane to achieve an average of 30% and 25% fewer bunches per vine than grower control (normal) crop load treatment, which had an average of 83 and 87 bunches per vine in 2020 and 2021, respectively. Normal crop load treatment vines were not adjusted for bunch number. In short, treatments were defined as deficit irrigation with low crop load (DL), deficit irrigation with normal crop load (DN), full irrigation with low crop load (FL), and full irrigation with normal crop load (FN).

### 3.4. Vine Physiology, Canopy, and Yield Measures

Vine leaves were placed in aluminium foil zip-lock bags 1 hr prior to measuring stem water potential (Ψ_S_) and leaf water potential (Ψ_L_) on neighbouring leaves. Measurements were taken using a Model 1505D Pressure Chamber (PMS Instrument Company, Albany, OR, USA). The rate of photosynthesis, stomatal conductance, and transpiration were analysed using an LI-6400/XT portable console (LI-COR Biosciences, Lincoln, NE, USA). Vine measurements were taken on three vines per treatment between 12:00 and 14:00 on days without cloud cover, which were 86, 97, and 108 dpf in 2020 and 75, 86, 97, 118, and 126 dpf in 2021. Viticanopy App [41] was used at 108 dpf to analyse the leaf area index (LAI) in 2020 and 2021 (*n* = 6 vines per treatment). Vine cane number, total cordon length, and cane weight were measured in 2021 (*n* = 10 vines). Yield components of vines (*n* = 10; vine bunch count and total yield weight) were obtained at the time of harvest in both vintages. The Ravaz index [42] was calculated from the average pruning weight per meter divided by yield per meter for vines under each treatment in 2020/2021.

### 3.5. Sampling and Berry Sorting

In 2020 and 2021, intact berries were sampled approximately every 10 days from early ripening. Sampling started at 76 dpf in 2020, followed by 86, 97, and 108 dpf with harvest at 111 dpf resulting in five sampling dates. In 2021, sampling occurred on 75, 87, 97, 108, 118, and 126 dpf, with harvest at 128 dpf resulting in seven sampling dates. Due to restrictions associated with the COVID-19 pandemic, the fruit had to be harvested prior to commercial harvest in 2020. The treatment vine sampling regime has been detailed previously [25]. In summary, berries were cut with the pedicel remaining intact from the top, middle, and bottom of bunches (*n* = 5 per position) to represent intra-bunch variability, and bunches (*n* = 7) were sampled randomly from the east and west facing side of each vine (*n* = 6 per treatment) to represent intra- and inter-vine variability. Grape samples were stored on ice before being transported to the laboratory and stored at 4 °C overnight. Separately for each vineyard treatment, berries were sorted at room temperature according to maturity class using NaCl salt density baths (*n* = 16) ranging from 1040 kg/m^3^ to 1152 kg/m^3^, with increments of 7 kg/m^3^ [43]. The population across each maturity class was recorded for every bunch. The sorted maturity classes were washed with distilled water and dried before being homogenised using an Ultra Turrax homogeniser (IKA T-18 Basic, IKA Works, Selangor, Malaysia) at 18,000 rpm for 2 min. Maturity classes for each treatment were subsampled into 1 g aliquots and stored at −20 °C before further analysis.

### 3.6. Vineyard Spatial Variation

In 2021, the Commercial Block (Figure 10) was sectioned based on soil electrical conductivity (EC_a_) data from EM38 (Geonics, Mississauga, ON, Canada) surveys conducted in 2010 and 2011 (Figure 10B). Between three and five healthy vines were sampled from each subsection to ensure representation of the spatial variability of the Commercial Block. Vines were sampled at 75, 108, and 128 dpf with the same sampling regime used for treatment vines and sorted into maturity classes as outlined in Section 3.5. These were georeferenced using a differentially corrected Global Navigation Satellite System (GNSS) accurate to approximately 20 cm in the *x* and *y* planes. The normalised difference vegetation index (NDVI) values were extracted from satellite imagery captured at 63 dpf with 80 cm resolution (Datafarming, Highfields, QLD, Australia). The image band containing non-vine pixels was replaced with null values so that only image bands with vine pixels were used to calculate NDVI. Values were scaled to 0–255 prior to extracting NDVI values relevant to the trial (Figure 10C); note that a separate scaling was used for two sub-blocks due to their different age. Yield components (*n* = 30 vines) and pruning weights (*n* = 12 vines) were measured at the time of harvest (128 dpf) and after leaf fall (June 2021), accordingly. Crop load was calculated as the Ravaz index, based on vine yield (kg) divided by vine pruning weight and averaged per treatment (*n* = 12).

### 3.7. Analysis of Grape Physical and Chemical Parameters

Measures of berry TSS, pH, malic and tartaric acids, methoxypyrazines, methyl cellulose precipitable tannins (MCP tannin), and absorbance at 520 nm were obtained from a previous study [25] and are briefly described below. Additional measurements of average berry FW and CIELab colour coordinates were undertaken for the current study. All measurements were conducted on the sorted maturity classes for the respective treatments and completed within six months of vineyard sampling.

#### 3.7.1. Average TSS, pH, and Berry FW

Average FW was determined using a Mettler Toledo analytical balance after berries had been sorted, washed, and dried. An aliquot of 1 g of fresh grape homogenate was centrifuged for 5 min at 1200× *g,* and TSS (°Brix) and pH of the supernatant were measured using a bench-top refractometer (Hanna Instruments, Woonsocket, RI, USA) and pH meter (OHAUS, Parsippany, NJ, USA).

#### 3.7.2. Total Tannin and Organic Acid Measurements

Grape homogenate total tannin concentrations were analysed using the methylcellulose precipitable tannin assay [44]. Malic and tartaric acid concentrations were measured by enzymatic assays (Megazyme, Bray, Ireland) with absorbance recorded on an Infinite 200 PRO UV-Vis spectrophotometer (Tecan Group Ltd., Männedorf, Switzerland). Tartaric acid values for samples collected 76 dpf and 86 dpf in 2020, and 75 dpf in 2021 were not obtained as the concentration was below the limit of detection (0.82 g/L) after samples had been diluted to remove interference from malic acid concentrations > 2.0 g/L. The dilution factor depended on the concentration of malic acid in the sample, which was determined prior to tartaric acid analysis.

#### 3.7.3. Methoxypyrazine Extraction and Quantification

Methoxypyrazines were extracted according to Dunlevy et al. [45] and analysed by stable isotope dilution assay with headspace SPME-GC-MS following the previously reported procedure [14], with the inclusion of deuterated internal standards d_3_-IPMP and d_3_-SBMP for quantification of IPMP and SBMP, respectively [21].

#### 3.7.4. Absorbance and CIELab Measures

Extracts of grape homogenates were prepared using 50% aqueous ethanol following the method previously detailed [44], and supernatants were diluted prior to analysis with a HORIBA Aqualog spectrophotometer (Aqualog-UV-800-C, Quark Photonics, Adelaide, SA, Australia) using a macro fluorescence cuvette (Sigma-Aldrich, Castle Hill, NSW, Australia) and a previously detailed method [25,46]. Absorbance at 520 nm (A520) and CIELab measures were collected using Aqualog software (version 4.2, HORIBA Instruments Inc., Irvine, CA, USA), and the colour index for red wine grapes (CIRWG) was computed following the previous method [34].

### 3.8. Data Analysis

The statistical software program R (R Foundation for Statistical Computing, Vienna, Austria) version 4.2.1 in RStudio (RStudio Inc., Boston, MA, USA) was used for analyses. The packages “stats”, “emmeans”, “lmerTest”, “ggplot2”, “dplyr”, “FactoMineR”, “corrplot”, “psych” and “multcomp” were used for data normalisation, log transformation, correlation matrix visualisation, multivariate analysis, data visualisation, and throughout data analyses.

#### 3.8.1. Treatment Trial Vine Data

Vine physiology, canopy, and yield parameters were compared using one-way ANOVA and pairwise comparisons of the estimated marginal means with Bonferroni adjustment (α = 0.05).

#### 3.8.2. Missing Values

Missing values for grape homogenate pH, IBMP, MCP tannin, A520, CIELab coordinates, and malic and tartaric acids were incurred at the extreme high and low maturity classes (≤7 berries from these density baths did not provide enough material for analyses). Such grape chemical values were deemed missing, not at random, and linear regression models were used to estimate the missing values. This method was used to estimate the chemical parameters of three maturity classes on average per sample date.

#### 3.8.3. Linear Mixed Models

Mixed effect linear regression models (i.e., linear mixed model, LMM) with the restricted maximum likelihood method were applied to determine vintage and sample date effects and crop load and irrigation interaction effects on grape chemical and physical properties. Berry measurements were set as dependent factors; vintage, sample date, crop load, and irrigation were set as the fixed effects, where appropriate, and block, vine, and bunch were set as nested random factors.

#### 3.8.4. Grape Chemical and Physical Data Mean and Residual Analysis

The absolute residuals were extracted from LMMs as a measure of variability [12], and estimates of the contribution of each random effect (block, vine, and bunch) to the variance of the dependent variable were recorded. Mean and absolute residual comparisons for vintage, sample date, and treatment were completed using one-way ANOVA, and two-way ANOVA was carried out for treatment interaction effects, followed by Bonferroni adjusted pairwise comparisons (α = 0.05). Principal component analysis (PCA) was conducted on log-transformed absolute residuals of TSS, FW, pH, malic acid, IBMP, MCP tannin, and A520, and average bunch values of each measure, separately for each vintage.

#### 3.8.5. Spatial Trial Vine Canopy and Size Attributes

Subsections of the Commercial Block based on EC_a_ values were classified using QGIS version 3.18.0 and Precision Agriculture Tools plugin [47]. Vine canopy architecture parameters, LAI, and yield parameters were scaled to have a mean of zero and standard deviation equal to one based on vine age prior to further analysis. LMMs were developed with TSS, FW, and pH as dependent factors, scaled vine canopy and yield parameters as fixed effects, and vine and bunch as nested random effects. The absolute residuals were then plotted against the appropriately scaled vine canopy or yield parameter, and the strength of the linear relationship was assessed using the Pearson correlation coefficient.

#### 3.8.6. Grape Heterogeneity Index

GHI score was calculated for heterogeneity level *i* (i.e., bunch, vine, or block) as the range of values for measurement *j* multiplied by the sum of absolute residuals *res* of measurement *j* as shown in Equation (1):

(1) $$GHI_{i}=\frac{1}{n}\left\{\left.\left((\max{\left(x\right)}_{ij}-\min{\left(x\right)}_{ij})\times \sum res_{ij}\right)+\left(\left(\max{\left(x\right)}_{ik}-\min{\left(x\right)}_{ik}\right)\times \sum res_{ik}\right)+\ldots \;\right\}\right.$$

where *n* is the number of measurements used in the calculation. Absolute residuals were extracted from LMMs and log-transformed and scaled using the min-max scaling method reported elsewhere [26]. An additive aggregation method of the range multiplied by the sum of residuals of measurements A520, FW, IBMP, malic acid, MCP tannin, pH, and TSS was used to calculate the GHI score, unless stated otherwise. An alternate calculation of GHI was used in the multimodelling approach in the uncertainty analysis of Equation (1), as shown in Equation (2):

(2) $$GHI_{i}^{*}=\frac{1}{n}\left\{\left.mean{\left(res\right)}_{ij}+mean{\left(res\right)}_{ik}+\ldots \;\right\}\right.$$

Comparisons between GHI score and the fixed effects of vintage, sample date, and treatment were completed using LMMs with block, vine, and bunch as nested random effects. One-way (vintage and sample date) and two-way (treatment) ANOVA with pairwise comparisons (α = 0.05, Bonferroni adjusted) were applied to mean GHI scores.

## 4. Conclusions

As a summary tool to describe overall grape heterogeneity, GHI has been proposed as a composite index that relies on absolute residuals of TSS, FW, pH, IBMP, malic acid, A520, and MCP tannin extracted from LMMs used to account for fixed and random effects. Indicator selection was derived from a thorough analysis of the trends in the residuals from grape maturity indices over multiple sampling dates and between viticultural treatments. This analysis gave transparency to the results obtained from implementing the GHI, which showed good sensitivity and robustness for analysing total grape heterogeneity. Although important to Cabernet Sauvignon as studied herein, it appeared that IBMP could be excluded, and the GHI would remain a useful measure of grape heterogeneity, thus broadening its applicability to cultivars that do not produce this varietal compound.

Further consideration could be given to geometric or multicriteria aggregation methods that could compensate samples with low residuals; in other words, ‘reward’ samples that have variability below a threshold for two or more indicators so that the range in GHI scores would be larger [26].

Based on GHI scores, variability decreased throughout ripening and plateaued when samples became more mature, highlighting that harvesting fruit too early (even if perhaps technologically mature) could result in the presence of high grape heterogeneity. There seemed to be minimal effects on the GHI score of the applied treatments, but the results from imposing water deficit with a normal crop load implied that stressed vines do not produce more variable fruit. A relationship between GHI and vine attributes appeared to be lacking, but the use of a simplified version of the GHI was consistent with observed trends between TSS, FW, and pH residuals and vine LAI, NDVI, pruning weight, vine yield, Ravaz index, and soil EC_a_. The potential of GHI scores for comparing overall grape heterogeneity in parcels of fruit was demonstrated, providing the first objective tool to assess this phenomenon. Further studies can be envisaged that build upon the GHI with other varieties and regions and utilise it to assess the effects of grape maturity variability on vineyard practices or wine chemistry and sensory characteristics.

## Data Availability

The data presented in this study are available in the article and Supplementary Materials.

## Supplementary Materials

The following supporting information can be downloaded at: https://www.mdpi.com/article/10.3390/plants12071442/s1, Figure S1. Bar charts showing estimated marginal means between 2019/2020 and 2020/2021; Figure S2. Bar charts showing the changes in the bunch-to-bunch variability; Figure S3. Estimated marginal means and residuals of CIRWG between 2019/2020 and 2020/2021; Figure S4. Estimated marginal means and residuals of tartaric acid between 2019/2020 and 2020/2021; Figure S5. Bar charts showing estimated marginal means of maturity indices between 2019/2020 and 2020/2021 in response to treatments; Figures S6 to S11. Scatter plots of the relationship between scaled leaf area index (LAI), vegetative growth, soil electrical conductivity (EC_a_), normalised difference vegetation index (NDVI), vine yield, and Ravaz Index values and total soluble solids and residuals, fresh weight and residuals, and pH and residuals; Table S1. Standard deviation and percentage of each variation source contributing to overall variability; Table S2. Canopy, yield, and vine balance parameters according to irrigation and crop load treatments; Table S3. Measurements of vine physiology responses to irrigation and crop load treatments on individual sampling dates; Table S4. Significance (*p*-values) of main and interaction effects of crop load (normal or low) and irrigation (deficit or full) on absolute residuals of grape maturity measures on individual sampling dates.
