# Supplementary material for: Grape Heterogeneity Index: Assessment of Overall Grape Heterogeneity Using an Aggregation of Multiple Indicators
Source: Plants (Basel). 2023 Mar 24;12(7):1442. doi: 10.3390/plants12071442 (PMC10097037; doi:10.3390/plants12071442)
Supplement: Supplementary file 1 [file plants-12-01442-s001.zip › plants-2281143-supplementary.pdf]

SUPPORTING INFORMATION FOR

Grape Heterogeneity Index: Assessment of Overall Grape Heterogeneity Using an Aggregation of Multiple Indicators

Claire E. J. Armstrong[1,2], Pietro Previtali[1,2], Paul K. Boss[1,3], Vinay Pagay[1,2], Robert G. V. Bramley[3], David W. Jeffery[1,2,*]

[1] Australian Research Council Training Centre for Innovative Wine Production, The University of Adelaide, PMB 1, Glen Osmond, South Australia 5064, Australia
[2] School of Agriculture, Food and Wine, and Waite Research Institute, The University of Adelaide, PMB 1, Glen Osmond, South Australia 5064, Australia
[3] CSIRO Agriculture and Food, Locked Bag 2, Glen Osmond, South Australia 5064, Australia
***** Correspondence: david.jeffery@adelaide.edu.au (D. W. Jeffery)

Table of Contents

	Page
**Figure S1.** Bar charts showing estimated marginal means between 2019/2020 and 2020/2021 of (A) absorbance at 520 nm (A520), (B) fresh weight (FW), (C) 3-isobutyl-2-methoxypyrazine (IBMP) concentration, (D) malic acid concentration, (E) methyl cellulose precipitable (MCP) tannin concentration, (F) pH, and (G) total soluble solids (TSS) according to mean ± SEM.	**S-2**
**Figure S2.** Bar charts showing the changes in the bunch-to-bunch variability between 2019/2020 and 2020/2021 of (A) absorbance at 520 nm (A520), (B) berry fresh weight (FW), (C) 3-isobutyl-2-methoxypyrazine (IBMP), (D) malic acid, (E) methyl cellulose (MCP) tannin, (F) pH, and (G) total soluble solids (TSS) according to mean of residuals ± SEM.	**S-2**
**Figure S3.** Estimated marginal means comparing CIRWG between (A) vintages 2019/2020 and 2020/2021, sample dates (days post flowering, dpf) in (B) 2019/2020, and (C) 2020/2021, and differences in CIRWG residuals between (D) vintages 2019/2020 and 2020/2021, and sample dates in (E) 20219/2020, and (F) 2020/2021 according to mean ± SEM.	**S-3**
**Figure S4.** Estimated marginal means comparing tartaric acid concentrations between (A) vintages 2019/2020 and 2020/2021, sample dates (days post flowering, dpf) in (B) 2019/2020, and (C) 2020/2021, and differences in tartaric acid residuals between (D) vintages 2019/2020 and 2020/2021, and sample dates in (E) 20219/2020, and (F) 2020/2021 according to mean ± SEM.	**S-3**
**Figure S5.** Bar charts showing estimated marginal means between 2019/2020 and 2020/2021 of (A, B) absorbance at 520 nm (A520), (C, D) berry fresh weight (FW), (E, F) 3-isobutyl-2-methoxypyrazine (IBMP) concentration, (G, H) malic acid concentration, (I, J) methyl cellulose precipitable (MCP) tannin concentration, (K, L) pH, and (M, N) total soluble solids (TSS) in response to crop load and irrigation regimes for each sample date (days post-flowering, dpf).	**S-4**
**Figures S6 to S11**. Scatter plots of the relationship between scaled leaf area index (LAI), vegetative growth, soil electrical conductivity (EC_a), normalised difference vegetation index (NDVI), vine yield, and Ravaz index values and (A, B) total soluble solids (TSS) and residuals, (C, D) berry fresh weight and residuals, and (E, F) pH and residuals of vines (n = 30) across the Commercial Block for different sampling dates (75, 108 and 128 dpf) in season 2020/2021.	**S-5-10**
**Table S1.** Standard deviation and percentage of each variation source contributing to overall variability for grape maturity measures on individual sampling dates.	**S-11**
**Table S2.** Canopy, yield, and vine balance parameters according to irrigation and crop load treatments.	**S-13**
**Table S3.** Measurements of vine physiology responses to irrigation and crop load treatments on individual sampling dates.	**S-14**
**Table S4.** Significance (p-values) of main and interaction effects of crop load (normal or low) and irrigation (deficit or full) on absolute residuals of grape maturity measures on individual sampling dates.	**S-16**

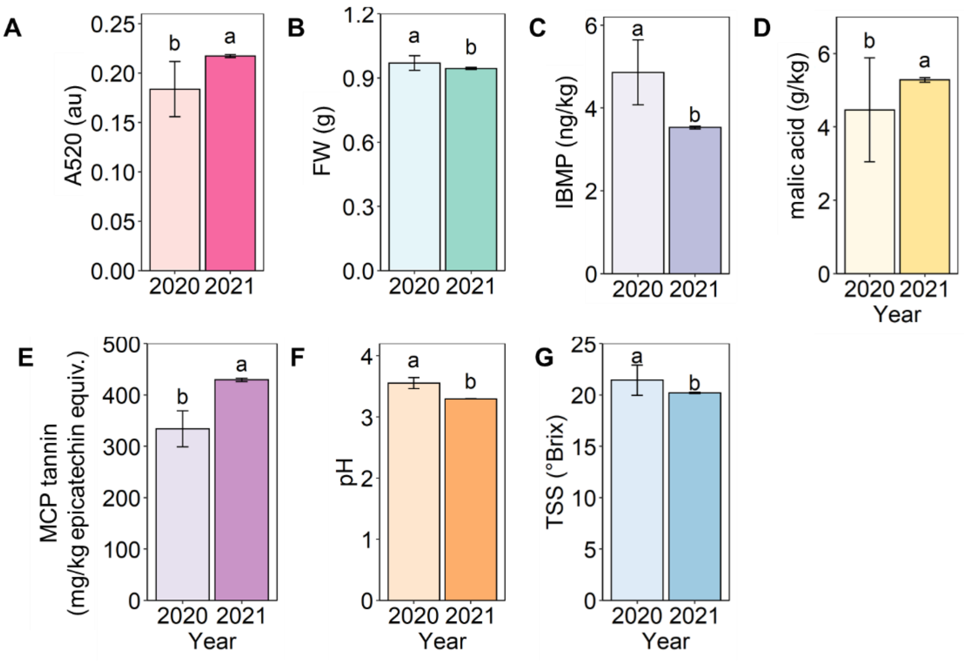

Figure S1. Bar charts showing estimated marginal means between 2019/2020 and 2020/2021 of (A) absorbance at 520 nm (A520), (B) fresh weight (FW), (C) 3-isobutyl-2-methoxypyrazine (IBMP) concentration, (D) malic acid concentration, (E) methyl cellulose precipitable (MCP) tannin concentration, (F) pH, and (G) total soluble solids (TSS) according to mean ± SEM. Different lower-case letters for a given measurement represent significant differences between vintage (linear mixed model, α = 0.05).

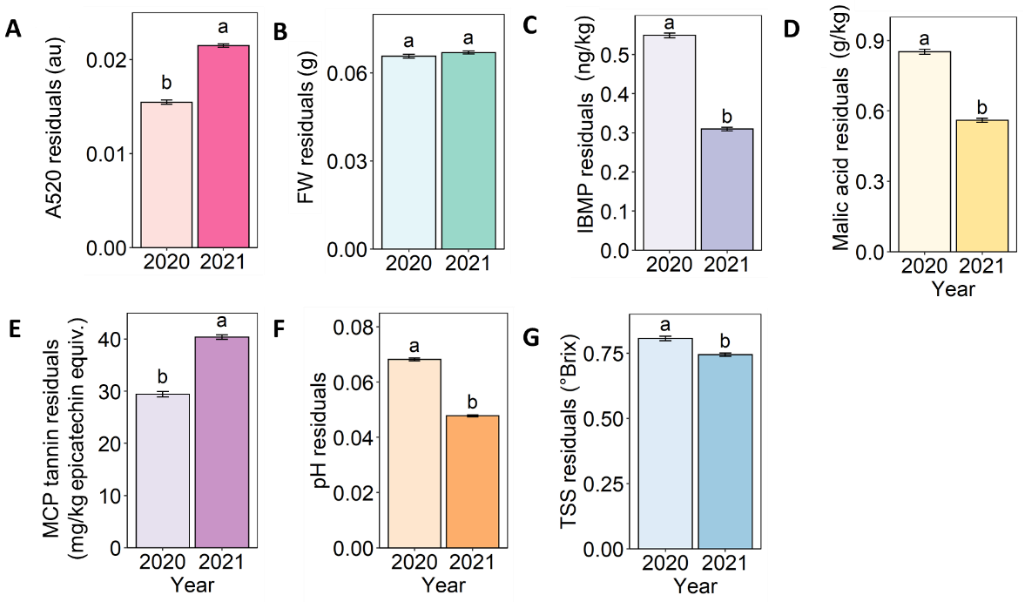

Figure S2. Bar charts showing the changes in the bunch-to-bunch variability between 2019/2020 and 2020/2021 of (A) absorbance at 520 nm (A520), (B) berry fresh weight (FW), (C) 3-isobutyl-2-methoxypyrazine (IBMP), (D) malic acid, (E) methyl cellulose (MCP) tannin, (F) pH, and (G) total soluble solids (TSS) according to mean of residuals ± SEM. Different lower-case letters for a given measurement represent significant differences between vintage (linear mixed model, α = 0.05).

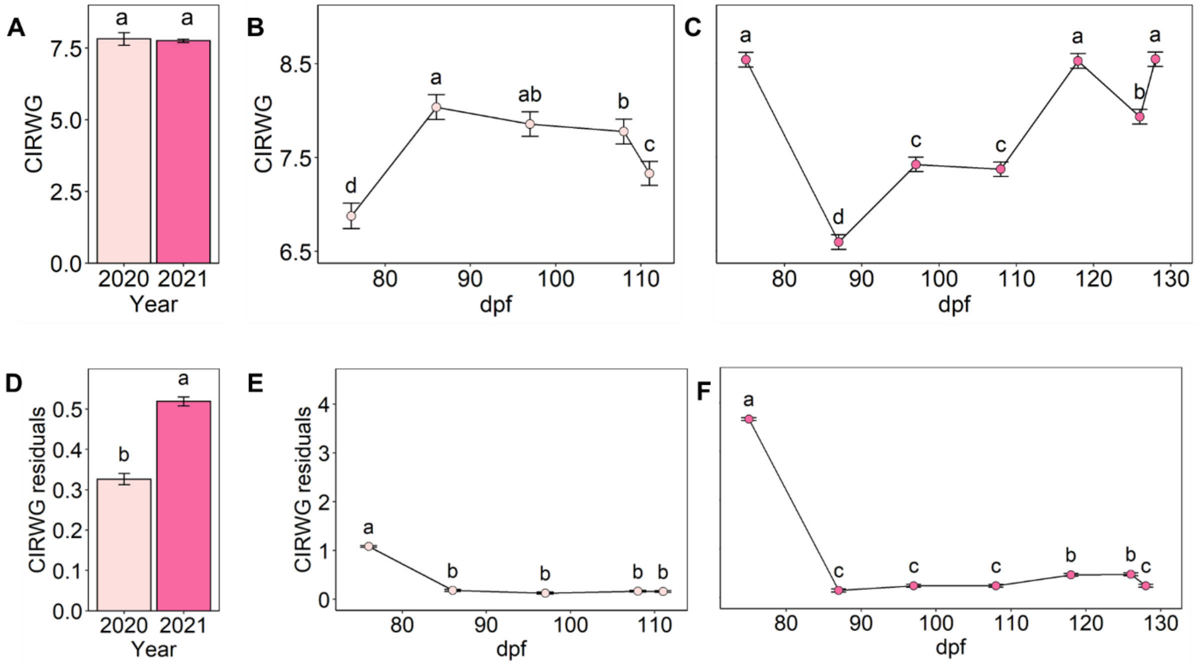

Figure S3. Estimated marginal means comparing CIRWG between (A) vintages 2019/2020 and 2020/2021, sample dates (days post flowering, dpf) in (B) 2019/2020, and (C) 2020/2021, and differences in CIRWG residuals between (D) vintages 2019/2020 and 2020/2021, and sample dates in (E) 20219/2020, and (F) 2020/2021 according to mean ± SEM. Different lower-case letters represent significant differences between vintage or sample date within a vintage (linear mixed model, α = 0.05, Bonferroni-adjusted).

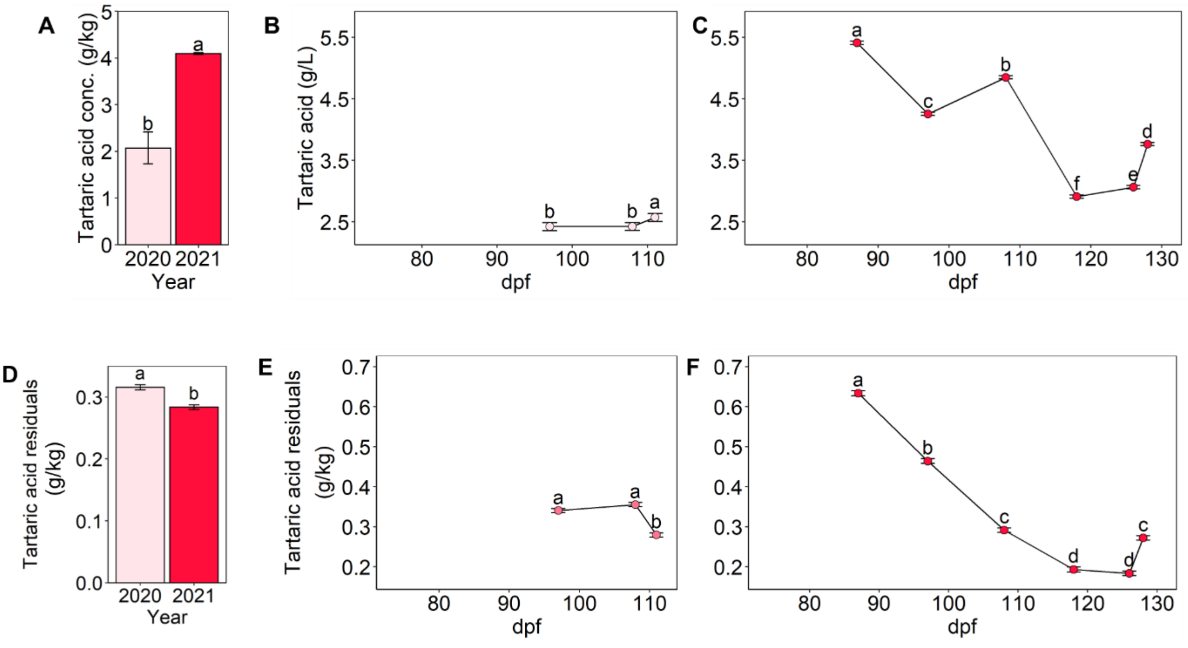

Figure S4. Estimated marginal means comparing tartaric acid concentrations between (A) vintages 2019/2020 and 2020/2021, sample dates (days post flowering, dpf) in (B) 2019/2020, and (C) 2020/2021, and differences in tartaric acid residuals between (D) vintages 2019/2020 and 2020/2021, and sample dates in (E) 20219/2020, and (F) 2020/2021 according to mean ± SEM. Different lower-case letters represent significant differences between vintage or sample date within a vintage (linear mixed model, α = 0.05, Bonferroni-adjusted).

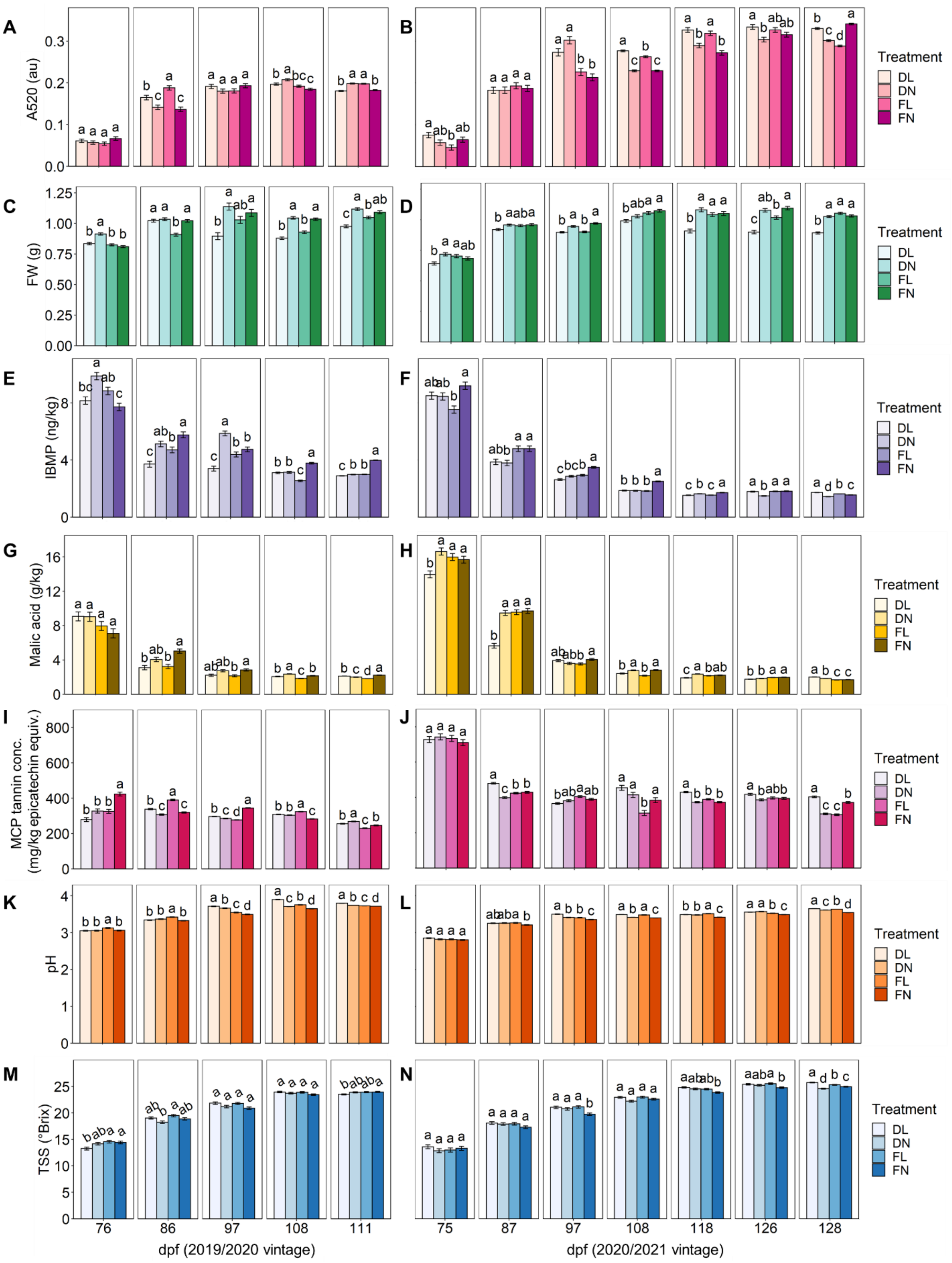

Figure S5. Bar charts showing estimated marginal means between 2019/2020 and 2020/2021 of (A, B) absorbance at 520 nm (A520), (C, D) berry fresh weight (FW), (E, F) 3-isobutyl-2-methoxypyrazine (IBMP) concentration, (G, H) malic acid concentration, (I, J) methyl cellulose precipitable (MCP) tannin concentration, (K, L) pH, and (M, N) total soluble solids (TSS) in response to crop load and irrigation regimes for each sample date (days post-flowering, dpf). Bars and error bars represent the means ± SEM (n = 6 vines per treatment). Different letters on a given sample date represent significant differences between treatments (linear mixed model, α = 0.05, Bonferroni-adjusted). DL = deficit irrigation/low crop load, DN = deficit irrigation/normal crop load, FL = full irrigation/low crop load, FN = full irrigation/normal crop load (grower control).

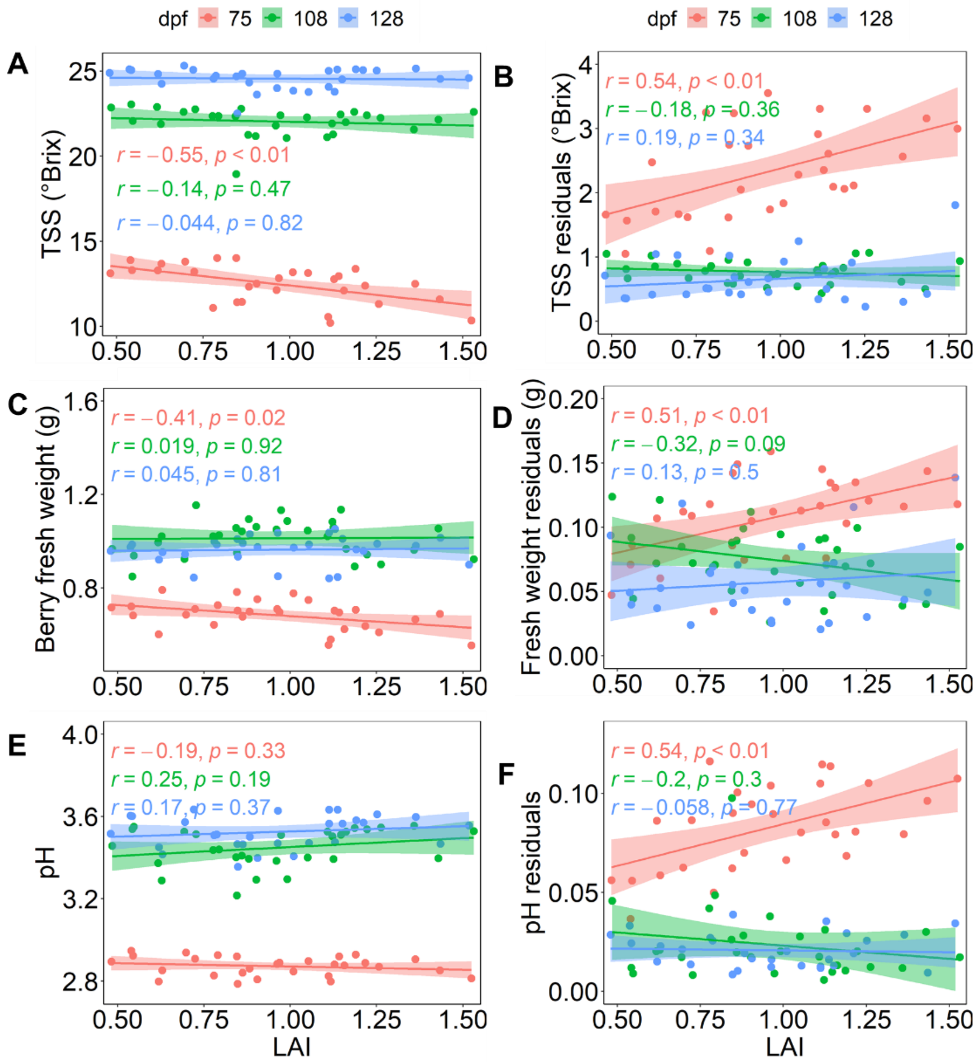

Figure S6. Scatter plots of the relationship between scaled leaf area index (LAI) values and (A, B) total soluble solids (TSS) and residuals, (C, D) berry fresh weight and residuals, and (E, F) pH and residuals of vines (n = 30) across the Commercial Block for different sampling dates (75, 108 and 128 dpf) in season 2020/2021. Linear fits (–) are graphed to aid visualisation of the relationship between variables, with Pearson correlation coefficient (r) and p-value indicating the strength and significance of the relationship.

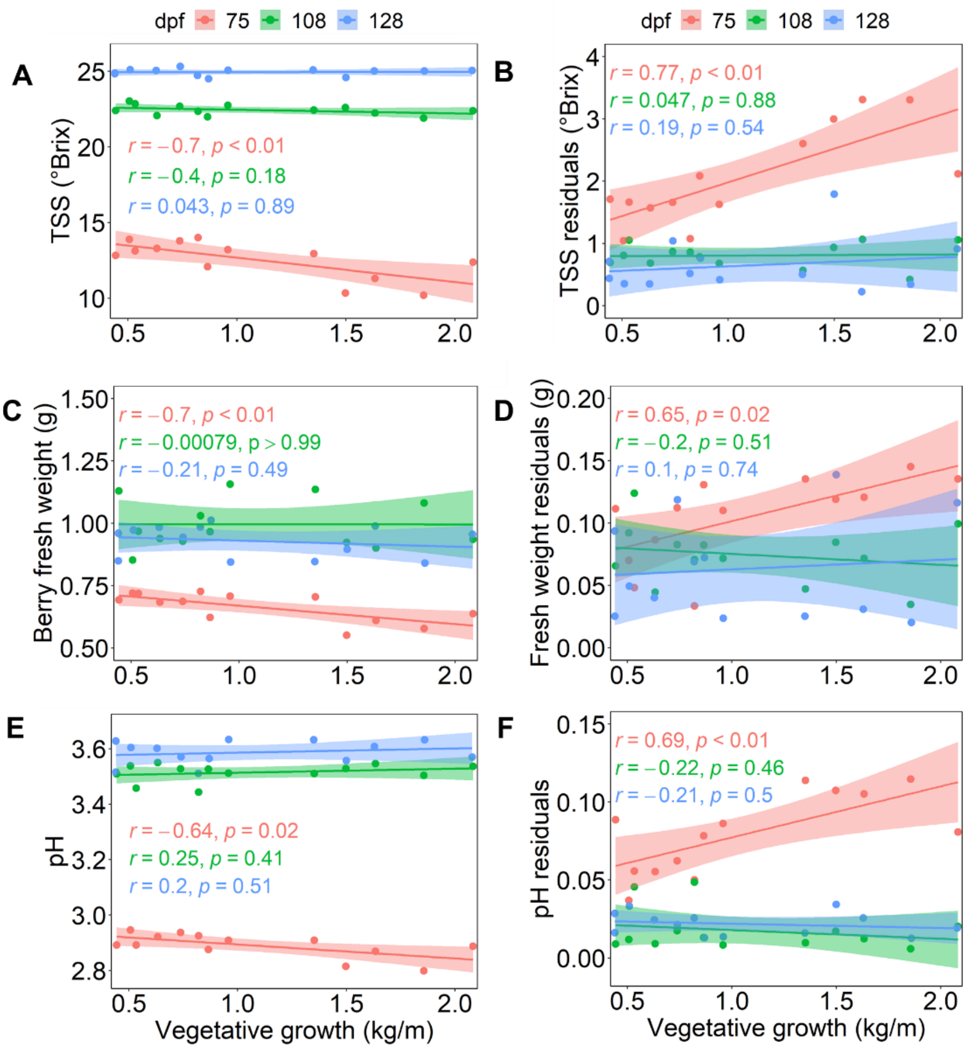

Figure S7. Scatter plots of the relationship between vine vegetative growth values and (A, B) total soluble solids (TSS) and residuals, (C, D) fresh weight and residuals, and (E, F) pH and residuals for vines (n = 13) across the Commercial Block for different sampling dates (75, 108 and 128 dpf) in season 2020/2021. Linear fits (–) are graphed to aid visualisation of the relationship between variables, with Pearson correlation coefficient (r) and p-value indicating the strength and significance of the relationship.

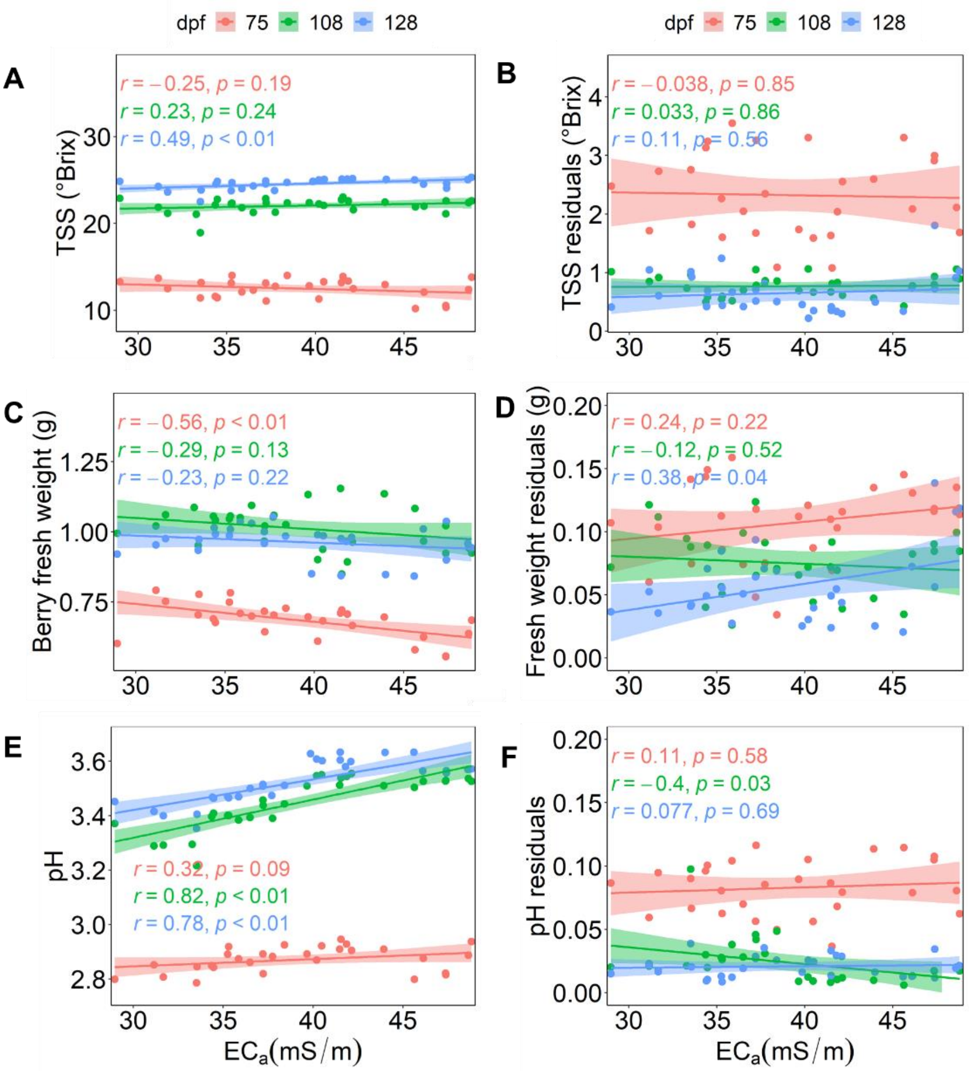

Figure S8. Scatter plots of the relationship between soil electrical conductivity (EC_a) values and (A, B) total soluble solids (TSS) and residuals, (C, D) fresh weight and residuals, and (E, F) pH and residuals of vines (n = 30) across the Commercial Block for different sampling dates (75, 108 and 128 dpf) in season 2020/2021. Linear fits (–) are graphed to aid visualisation of the relationship between variables, with Pearson correlation coefficient (r) and p-value indicating the strength and significance of the relationship.

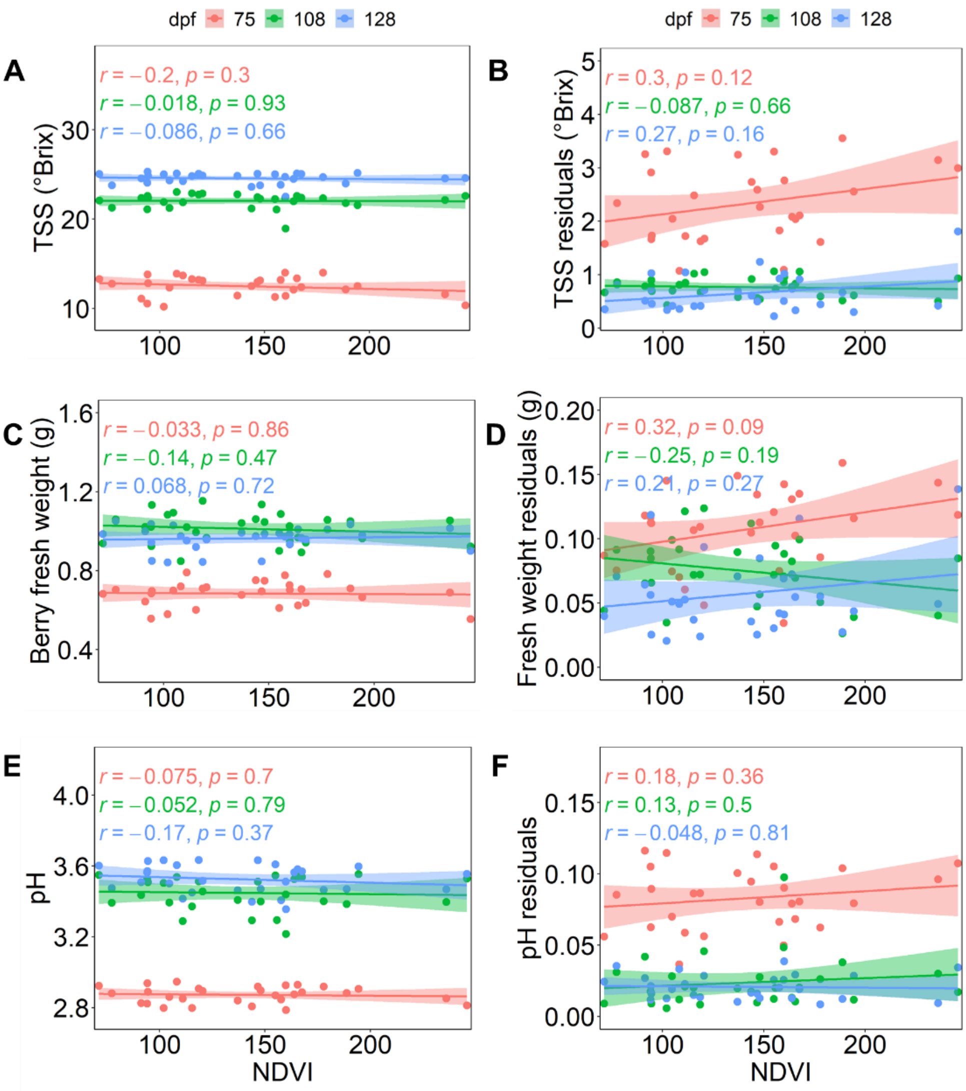

Figure S9. Scatter plots of the relationship between scaled vine normalised difference vegetation index (NDVI) values and (A, B) total soluble solids (TSS) and residuals, (C, D) fresh weight and residuals, and (E, F) pH and residuals for vines (n = 30) across the Commercial Block for different sampling dates (75, 108 and 128 dpf) in season 2020/2021. Linear fits (–) are graphed to aid visualisation of the relationship between variables, with Pearson correlation coefficient (r) and p-value indicating the strength and significance of the relationship.

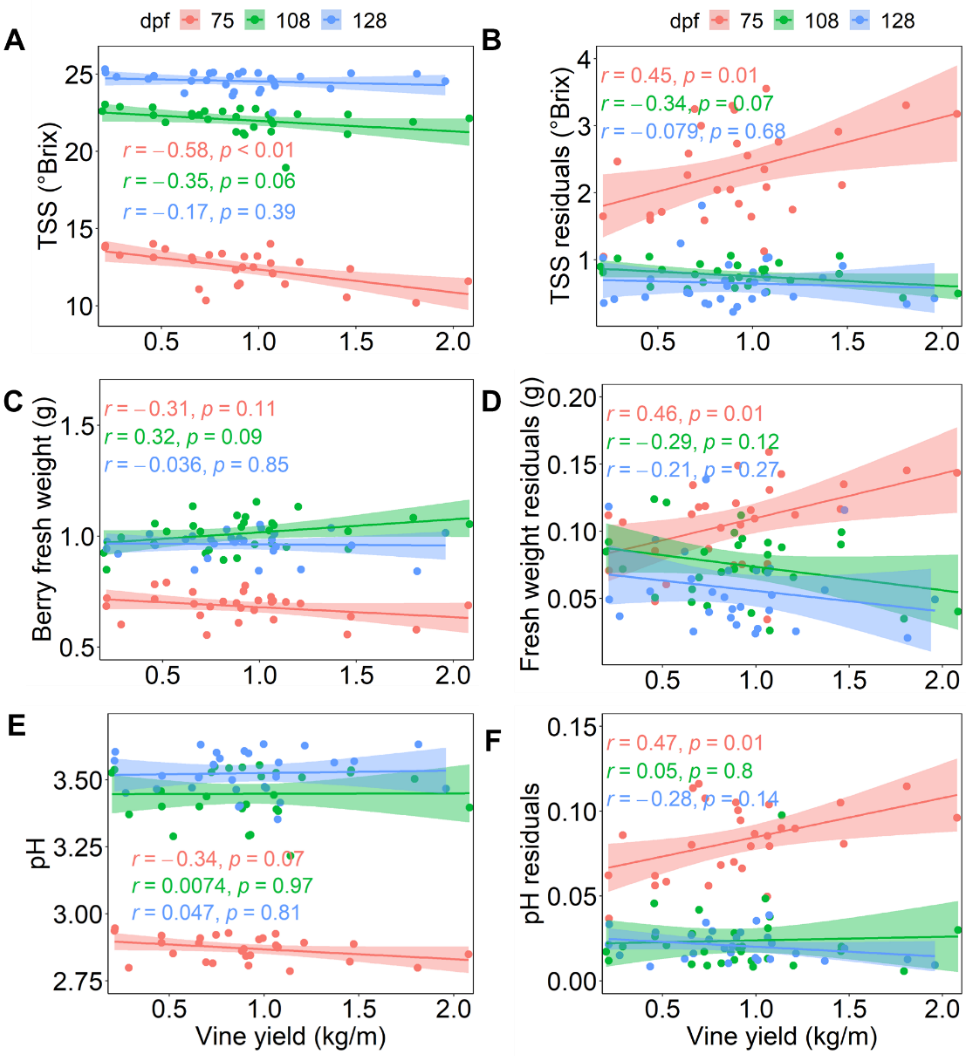

Figure S10. Scatter plots of the relationship between scaled vine yield values and (A, B) total soluble solids (TSS) and residuals, (C, D) fresh weight and residuals, and (E, F) pH and residuals for vines (n = 30) across the Commercial Block for different sampling dates (75, 108 and 128 dpf) in season 2020/2021. Linear fits (–) are graphed to aid visualisation of the relationship between variables, with Pearson correlation coefficient (r) and p-value indicating the strength and significance of the relationship.

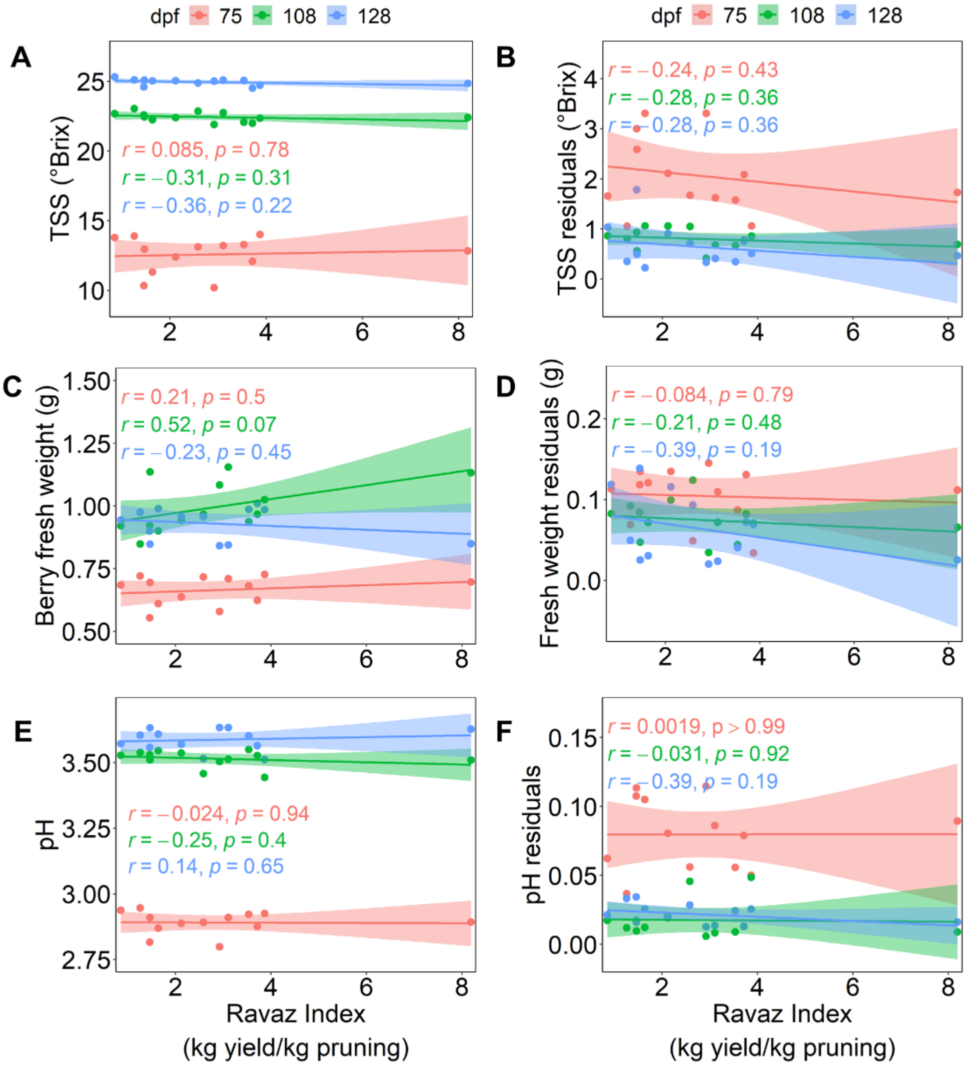

Figure S11. Scatter plots of the relationship between Ravaz index values and (A, B) total soluble solids (TSS) and residuals, (C, D) fresh weight and residuals, and (E, F) pH and residuals for vines (n = 13) across the Commercial Block for different sampling dates (75, 108 and 128 dpf) in season 2020/2021. Linear fits (–) are graphed to aid visualisation of the relationship between variables, with Pearson correlation coefficient (r) and p-value indicating the strength and significance of the relationship.

Table S1. Standard deviation and percentage of each variation source contributing to overall variability for grape maturity measures on individual sampling dates.[a]

			A520		TSS		FW		pH		Malic acid		IBMP		MCP tannin		Tartaric acid		CIRWG	
**Year**	**dpf**	**Variation source**	**SD**	**%**	**SD**	**%**	**SD**	**%**	**SD**	**%**	**SD**	**%**	**SD**	**%**	**SD**	**%**	**SD**	**%**	**SD**	**%**
2020	76	Bunch	0.018	37	1.3	37	0.052	37	0.052	35	2.5	36	1.2	35	45	27	NA	NA	1.2	30
		Vine	0.0072	15	0.38	11	0.0051	4	0.020	14	0.80	11	0.4	12	10	6	NA	NA	0.35	9
		Block	0.0	0.0	0	0	0	0	0	0	0	0	0	0	12	7	NA	NA	0	0
		Residuals	0.024	48	1.8	52	0.085	60	0.076	51	3.6	52	1.9	54	100	60	NA	NA	2.4	61
	86	Bunch	0.021	36	0.85	34	0.060	35	0.033	28	0.80	27	0.67	33	16	24	NA	NA	0.17	33
		Vine	0.0098	16	0.44	17	0.012	7	0.017	15	0.51	17	0.41	20	6	8	NA	NA	0.052	10
		Block	0.0	0	0	0	0.0054	3	0	0	0	0	0	0	0	0	NA	NA	0.039	8
		Residuals	0.029	48	1.2	49	0.092	55	0.067	57	1.6	55	0.93	46	46	68	NA	NA	0.26	49
	97	Bunch	0.012	26	0.56	28	0.051	25	0.022	23	0.27	23	0.29	22	0.99	3	0.11	19	0.083	26
		Vine	0.0055	12	0.19	9.2	0.010	5	0.0090	10	0.11	10	0.092	7	0	0	0.040	7	0.025	8
		Block	0.0069	15	0.34	17	0.047	23	0.015	16	0.20	18	0.28	21	0	0	0	0	0.062	19
		Residuals	0.021	46	0.93	46	0.094	46	0.048	51	0.57	49	0.66	50	36	97	0.43	74	0.15	47
	108	Bunch	0.0070	24	0.41	25	0.028	21	0.0073	15	0.12	24	0.17	21	3.1	12	0.14	23	0.058	17
		Vine	0.0052	17	0.32	20	0.021	16	0.0081	17	0.088	17	0.12	15	2.2	8	0.035	6	0.053	16
		Block	0	0	0	0	0	0	0	0	0	0	0	0	0	0	0	0	0	0
		Residuals	0.017	59	0.90	55	0.082	63	0.033	68	0.30	58	0.51	64	21	80	0.44	72	0.22	67
	111	Bunch	0.0036	15	0.41	28	0.048	30	0.0049	13	0.073	22	0.042	10	6.2	18	0.054	12	0.045	18
		Vine	0	0	0.096	6.5	0.0088	6	0	0	0.017	5	0	0	4.2	12	0	0	0	0
		Block	0	0	0	0	0	0	0	0	0	0	0	0	0	0	0	0	0	0
		Residuals	0.020	85	0.97	66	0.10	64	0.033	87	0.23	72	0.38	90	23	69	0.38	88	0.21	82
2021	75	Bunch	0.025	35	1.7	37	0.055	12	0.052	35	1.7	34	1.1	35	68	31	NA	NA	3.46	35
		Vine	0.012	17	0.66	15	0.22	48	0.021	14	0.73	15	0.46	15	32	14	NA	NA	1.1	11
		Block	0	0	0	0	0	0	0	0	0	0	0	0	0	0	NA	NA	0	0
		Residuals	0.034	48	2.2	48	0.18	40	0.074	50	2.5	51	1.5	49	122	55	NA	NA	5.2	53
	87	Bunch	0.026	30	0.80	28	0.043	29	0.034	28	1.0	31	0.42	26	22	29	0.20	17	0.10	30
		Vine	0.013	15	0.39	14	0.0096	6	0.016	13	0.36	11	0.22	13	7	9	0.090	8	0.047	14
		Block	0.0053	6.2	0.23	8.1	0	0	0.0093	8	0.29	9	0.27	16	0	0	0.12	10	0	0
		Residuals	0.041	48	1.4	50	0.097	65	0.062	51	1.7	50	0.73	44	49	63	0.76	65	0.19	57
	97	Bunch	0.024	29	0.66	26	0.031	26	0.022	26	0.42	24	0.19	25	9.4	12	0.19	21	0.13	27
		Vine	0.018	22	0.53	21	0.0061	5	0.015	17	0.24	14	0.12	15	10	13	0.15	17	0.010	21
		Block	0	0	0	0	0	0	0.0047	5	0	0	0	0	3.6	5	0	0	0	0
		Residuals	0.040	49	1.4	53	0.080	68	0.044	51	1.1	63	0.46	60	57	71	0.57	63	0.24	52
	108	Bunch	0.011	22	0.79	34	0.054	31	0.012	26	0.21	30	0.10	17	21	12	0.25	37	0.058	23
		Vine	0.00084	1.7	0.19	8	0.018	10	0.0065	14	0.025	4	0.049	8	18	11	0.027	4	0.020	8
		Block	0	0	0.13	6.2	0	0	0	0	0.070	10	0	0	0	0	0	0	0.025	10
		Residuals	0.037	76	1.2	52	0.10	59	0.029	60	0.40	56	0.45	75	136	78	0.40	59	0.15	60

Table S1. Continued

			A520		TSS		FW		pH		Malic acid		IBMP		MCPT		Tartaric acid		CIRWG	
**Year**	**dpf**	**Variation source**	**SD**	**%**	**SD**	**%**	**SD**	**%**	**SD**	**%**	**SD**	**%**	**SD**	**%**	**SD**	**%**	**SD**	**%**	**SD**	**%**
2021	118	Bunch	0.011	20	0.29	21	0.038	23	0.0065	16	0.062	19	0.028	17	10	18	0.053	19	0.044	20
		Vine	0.0084	16	0.26	18	0.027	16	0.0036	9	0.056	17	0.022	13	2.8	5	0.033	12	0.024	11
		Block	0.0067	13	0.16	12	0.017	10	0.0070	17	0.035	11	0.018	11	5.3	10	0	0	0.030	14
		Residuals	0.026	51	0.68	49	0.084	50	0.023	58	0.17	53	0.097	59	34	66	0.20	69	0.12	54
	126	Bunch	0.014	22	0.40	24	0.034	20	0.010	13	0.059	24	0.072	24	13	23	0.10	24	0.050	21
		Vine	0.012	19	0.33	20	0.020	12	0.0060	7	0.056	22	0.067	22	13	24	0.071	17	0.037	15
		Block	0	0	0	0	0.017	10	0.0061	8	0	0	0	0	0	0	0	0	0.0010	4
		Residuals	0.037	59	0.91	56	0.10	59	0.059	72	0.13	54	0.16	54	28	53	0.25	59	0.15	60
	128	Bunch	0.0097	23	0.35	23	0.034	25	0.0076	25	0.03	18	0.043	24	12	17	0.14	21	0.048	21
		Vine	0.0022	5.0	0.12	8	0.013	10	0.00079	3	0.020	12	0.018	10	10	15	0.025	4	0.017	8
		Block	0	0	0	0	0	0	0	0	0	0	0	0	0	0	0	0	0	0
		Residuals	0.031	72	1.1	70	0.090	66	0.022	73	0.11	70	0.12	65	47	68	0.48	75	0.16	71

[a]Standard deviation (SD) values are from linear mixed models with crop load (normal and low) and irrigation (deficit and full) treatments as fixed effects and block, vine and bunch as random effects. A520, absorbance at 520 nm; TSS, total soluble solids; FW, berry fresh weight; IBMP, 3-isobutyl-2-methoxypyrazine; MCPT, methyl cellulose precipitable tannin; CIRWG, colour index for red wine grapes); dpf, days-post-flowering.

Table S2. Canopy, yield, and vine balance parameters according to irrigation and crop load treatments.[a]

Vintage	Treatment	LAI	Pruning weight (kg/vine)	Yield (kg/vine)	Bunches per vine	Bunch weight (g)	RI (kg yield/ kg pruning)
2019/2020	Crop load						
	Normal	2.15 ± 0.15 b	-	2.92 ± 0.33	82.7 ± 6.9 a	34.5 ± 2.8	-
	Low	2.49 ± 0.16 a	-	2.05 ± 0.33	58.4 ± 6.9 b	34.9 ± 2.8	-
	Irrigation						
	Full	2.59 ± 0.14 a	-	2.64 ± 0.33	75.0 ± 6.9	34.7 ± 2.8	-
	Deficit	2.06 ± 0.16 b	-	2.33 ± 0.33	66.1 ± 6.9	34.7 ± 2.8	-
	CL × Irr	ns		ns	ns	ns	
2020/2021	Crop load						
	Normal	2.30 ± 0.12	2.09 ± 0.15	5.88 ± 0.54 a	87.4 ± 7.7 a	68.3 ± 4.3	3.28 ± 0.30 a
	Low	2.08 ± 0.12	1.88 ± 0.15	4.24 ± 0.54 b	64.9 ± 7.7 b	64.7 ± 4.3	2.13 ± 0.30 b
	Irrigation						
	Full	2.32 ± 0.12	2.16 ± 0.15	5.57 ± 0.54	72.8 ± 7.7	71.2 ± 4.3	2.67 ± 0.30
	Deficit	2.06 ± 0.11	1.81 ± 0.15	4.54 ± 0.54	79.5 ± 6.9	61.8 ± 4.3	2.74 ± 0.30
	CL × Irr	ns	ns	ns	ns	ns	ns
Vintage		ns	-	***	ns	***	-

[a]Values are mean ± SEM of leaf area index (LAI, n = 5), and vine pruning weight and yield (n = 10), with different lower-case letters within a column indicating a significant difference between means within a vintage year, crop load, and irrigation ($p \leq 0.05$, one-way ANOVA). Interaction effects of crop load and irrigation (CL × Irr) were deduced from two-way ANOVA within a vintage, and vintage simple main effects were deduced by one-way ANOVA, *** denotes $p \leq 0.001$, ** denotes $p \leq 0.01$, * denotes ≤ 0.05, and ns denotes $p > 0.05$. LAI, leaf area index.

Table S3. Measurements of vine physiology responses to irrigation and crop load treatments on individual sampling dates.[a]

Vintage	dpf	Treatment	ΨL (MPa)	ΨS (MPa)	Assimilation (mmol/cm^3/s)	Stomatal conductance (mol/m^2/s)	Transpiration (mol/m^2/s)
2019/2020	86	Crop load					
		Normal	-1.27 ± 0.05	-1.23 ± 0.05	14.5 ± 0.6	0.15 ± 0.02	2.83 ± 0.21
		Low	-1.30 ± 0.05	-1.24 ± 0.05	13.7 ± 0.5	0.16 ± 0.02	2.93 ± 0.19
		Irrigation					
		Deficit	-1.46 ± 0.05 b	-1.40 ± 0.05 b	11.1 ± 0.6 b	0.10 ± 0.02 b	1.98 ± 0.21 b
		Full	-1.11 ± 0.05 a	-1.07 ± 0.05 a	17.1 ± 0.5 a	0.22 ± 0.02 a	3.79 ± 0.19 a
		CL × Irr	ns	ns	ns	ns	ns
	97	Crop load					
		Normal	-1.25 ± 0.03a	-1.26 ± 0.07	14.3 ± 0.6	0.17 ± 0.01	2.43 ± 0.14
		Low	-1.36 ± 0.03b	-1.20 ± 0.07	14.9 ± 0.6	0.19 ± 0.01	2.70 ± 0.14
		Irrigation					
		Deficit	-1.48 ± 0.03b	-1.36 ± 0.07b	12.1 ± 0.6 b	0.12 ± 0.01 b	2.10 ± 0.14 b
		Full	-1.13 ± 0.03a	-1.10 ± 0.07a	17.1 ± 0.6 a	0.24 ± 0.01 a	3.03 ± 0.14 a
		CL × Irr	***	ns	**	**	**
	108	Crop load					
		Normal	-1.20 ± 0.04	-0.95 ± 0.04	12.3 ± 0.6	0.15 ± 0.01a	2.85 ± 0.15
		Low	-1.18 ± 0.04	-1.06 ± 0.04	11.7 ± 0.7	0.12 ± 0.01b	2.75 ± 0.16
		Irrigation					
		Deficit	-1.25 ± 0.04	-1.09 ± 0.04	9.95 ± 0.68 b	0.09 ± 0.01 b	2.29 ± 0.16 b
		Full	-1.13 ± 0.04	-0.93 ± 0.04	14.1 ± 0.6 a	0.18 ± 0.01 a	3.31 ± 0.15 a
		CL × Irr	ns	**	ns	**	ns
2020/2021	75	Crop load					
		Normal	-1.49 ± 0.03	-1.18 ± 0.05	12.6 ± 0.6	0.14 ± 0.01	2.90 ± 0.20
		Low	-1.45 ± 0.03	-1.09 ± 0.05	12.3 ± 0.6	0.13 ± 0.01	2.76 ± 0.21
		Irrigation					
		Deficit	-1.55 ± 0.03b	-1.27 ± 0.05b	10.0 ± 0.6 b	0.08 ± 0.01 b	1.95 ± 0.21 b
		Full	-1.39 ± 0.03a	-1.00 ± 0.05a	14.9 ± 0.6 a	0.19 ± 0.01 a	3.71 ± 0.20 a
		CL × Irr	ns	ns	ns	ns	ns
	86	Crop load					
		Normal	-1.36 ± 0.03	-1.03 ± 0.03	12.4 ± 0.7	0.16 ± 0.01	3.51 ± 0.22
		Low	-1.42 ± 0.04	-0.98 ± 0.03	11.9 ± 0.8	0.15 ± 0.01	3.39 ± 0.23
		Irrigation					
		Deficit	-1.38 ± 0.03	-1.07 ± 0.03 b	9.94 ± 0.74 b	0.11 ± 0.01 b	2.68 ± 0.22 b
		Full	-1.40 ± 0.04	-0.94 ± 0.03 a	14.5 ± 0.8 a	0.20 ± 0.01 a	4.22 ± 0.23 a
		CL × Irr	ns	ns	*	*	*
	97	Crop load					
		Normal	-1.11 ± 0.06	-0.84 ± 0.03	-	-	-
		Low	-1.00 ± 0.06	-0.84 ± 0.04	-	-	-
		Irrigation					
		Deficit	-1.01 ± 0.06	-0.90 ± 0.04	-	-	-
		Full	-1.09 ± 0.06	-0.79 ± 0.03	-	-	-
		CL × Irr	ns	ns			
	118	Crop load					
		Normal	-1.58 ± 0.02 b	-1.47 ± 0.04	7.48 ± 0.68	0.07 ± 0.02	2.07 ± 0.30
		Low	-1.48 ± 0.02 a	-1.40 ± 0.04	7.64 ± 0.68	0.08 ± 0.02	2.31 ± 0.29
		Irrigation					
		Deficit	-1.63 ± 0.02 b	-1.56 ± 0.04b	4.21 ± 0.68 b	0.03 ± 0.02 b	1.10 ± 0.30 b
		Full	-1.43 ± 0.02 a	-1.30 ± 0.04a	10.9 ± 0.7 a	0.12 ± 0.02 a	3.28 ± 0.29 a
		CL × Irr	ns	ns	ns	ns	ns

Table S3. Continued

Vintage	dpf	Treatment	Ψ_L (MPa)	Ψ_S (MPa)	Assimilation (mmol/cm^3/s)	Stomatal conductance (mol/m^2/s)	Transpiration (mol/m^2/s)
	126	Crop load					
		Normal	-1.13 ± 0.03	-0.99 ± 0.06	9.63 ± 0.68	0.15 ± 0.02	2.09 ± 0.15
		Low	-1.08 ± 0.03	-0.88 ± 0.06	9.68 ± 0.68	0.14 ± 0.02	2.02 ± 0.15
		Irrigation					
		Deficit	-0.95 ± 0.03 a	-0.84 ± 0.06	7.62 ± 0.68	0.10 ± 0.02 b	1.56 ± 0.15 b
		Full	-1.26 ± 0.03 b	-1.03 ± 0.06	11.7 ± 0.7	0.19 ± 0.02 a	2.54 ± 0.15 a
		CL × Irr	***	ns	ns	ns	ns

[a]Values are means ± SEM of leaf water potential (Ψ_L, n = 6 per treatment), stem water potential (Ψs, n = 3 per treatment), and photosynthetic rate of assimilation (assim.), stomatal conductance and transpiration (n = 3 per treatment), vintages and sample dates were analysed separately, and values followed by different letter within a column are significantly different ($p \leq 0.05$, one-way ANOVA). Interaction effects of crop load and irrigation (CL × Irr) were deduced from two-way ANOVA, *** denotes $p \leq 0.001$, ** denotes $p \leq 0.01$, * denotes ≤ 0.05, and ns denotes $p > 0.05$.

Table S4. Significance (p-values) of main and interaction effects of crop load (normal or low) and irrigation (deficit or full) on absolute residuals of grape maturity measures on individual sampling dates.[a]

Year	dpf	Main and Interaction Effect	A520 p-value		TSS p-value		FW p-value		pH p-value		Malic acid p-value		IBMP p-value		MCPT p-value		Tartaric acid p-value		CIRWG p-value	
		CL	0.28	ns	0.063	ns	< 0.001	***	0.15	ns	0.050	*	< 0.001	***	0.041	*	-	-	< 0.001	***
	76	Irr	< 0.001	***	< 0.001	***	< 0.001	***	< 0.001	***	0.37	ns	< 0.001	***	< 0.001	***	-	-	< 0.001	***
		CL × Irr	0.0032	**	0.16	ns	< 0.001	***	< 0.001	***	< 0.001	***	0.64	ns	< 0.001	***	-	-	< 0.001	***
		CL	< 0.001	***	< 0.001	***	< 0.001	***	0.47	ns	< 0.001	***	< 0.001	***	0.35	ns	-	-	< 0.001	***
	86	Irr	< 0.001	***	0.702	ns	< 0.001	***	< 0.001	***	0.24	ns	0.0014	**	0.27	ns	-	-	0.12	ns
		CL × Irr	0.17	ns	0.198	ns	< 0.001	***	0.087	ns	< 0.001	***	< 0.001	***	< 0.001	***	-	-	0.054	ns
		CL	< 0.001	***	0.087	ns	< 0.001	***	< 0.001	***	< 0.001	***	< 0.001	***	< 0.001	***	< 0.001	***	< 0.001	***
2020	97	Irr	< 0.001	***	0.029	*	< 0.001	***	< 0.001	***	0.66	ns	< 0.001	***	< 0.001	***	< 0.001	***	< 0.001	***
		CL × Irr	0.80	ns	0.039	*	< 0.001	***	0.37	ns	0.19	ns	< 0.001	***	< 0.001	***	< 0.001	***	< 0.001	***
		CL	< 0.001	***	< 0.001	***	< 0.001	***	< 0.001	***	0.012	*	< 0.001	***	< 0.001	***	< 0.001	***	< 0.001	***
	108	Irr	< 0.001	***	< 0.001	***	< 0.001	***	< 0.001	***	< 0.001	***	0.0039	**	0.0062	**	< 0.001	***	0.54	ns
		CL × Irr	< 0.001	***	0.86	ns	0.41	ns	0.0019	**	< 0.001	***	< 0.001	***	< 0.001	***	< 0.001	***	0.19	ns
		CL	< 0.001	***	< 0.001	***	0.95	ns	< 0.001	***	0.27	ns	< 0.001	***	< 0.001	***	< 0.001	***	0.025	*
	111	Irr	0.80	ns	0.0060	**	0.0069	**	< 0.001	***	0.86	ns	< 0.001	***	0.0015	**	< 0.001	***	< 0.001	***
		CL × Irr	< 0.001	***	0.54	ns	< 0.001	***	< 0.001	***	0.052	ns	< 0.001	***	< 0.001	***	< 0.001	***	< 0.001	***
		CL	< 0.001	***	0.93	ns	0.018	*	< 0.001	***	< 0.001	***	< 0.001	***	0.37	ns	-	-	< 0.001	***
	75	Irr	< 0.001	***	0.47	ns	0.59	ns	< 0.001	***	0.044	*	0.71	ns	0.011	*	-	-	< 0.001	***
		CL × Irr	< 0.001	***	0.90	ns	0.68	ns	0.29	ns	< 0.001	***	0.25	ns	< 0.001	***	-	-	0.068	ns
		CL	< 0.001	***	0.048	*	< 0.001	***	< 0.001	***	< 0.001	***	< 0.001	***	< 0.001	***	< 0.001	***	< 0.001	***
	87	Irr	0.30	ns	0.016	*	< 0.001	***	0.094	ns	0.0030	**	< 0.001	***	0.15	ns	< 0.001	***	0.030	*
		CL × Irr	0.72	ns	0.20	ns	< 0.001	***	< 0.001	***	< 0.001	***	0.17	ns	0.23	ns	< 0.001	***	0.012	*
		CL	< 0.001	***	< 0.001	***	< 0.001	***	< 0.001	***	0.0068	**	0.18	ns	< 0.001	***	< 0.001	***	< 0.001	***
	97	Irr	< 0.001	***	< 0.001	***	< 0.001	***	< 0.001	***	< 0.001	***	< 0.001	***	0.64	ns	< 0.001	***	< 0.001	***
		CL × Irr	0.29	ns	< 0.001	***	< 0.001	***	< 0.001	***	< 0.001	***	< 0.001	***	< 0.001	***	< 0.001	***	< 0.001	***
		CL	0.47	ns	< 0.001	***	< 0.001	***	0.26	ns	< 0.001	***	< 0.001	***	< 0.001	***	< 0.001	***	0.28	ns
2021	108	Irr	0.37	ns	0.0013	**	0.45	ns	< 0.001	***	< 0.001	***	0.21	ns	< 0.001	***	< 0.001	***	< 0.001	***
		CL × Irr	0.11	ns	0.35	ns	0.15	ns	< 0.001	***	0.019	*	0.0083	**	0.38	ns	< 0.001	***	< 0.001	***
		CL	< 0.001	***	0.23	ns	0.75	ns	< 0.001	***	< 0.001	***	< 0.001	***	< 0.001	***	< 0.001	***	0.060	ns
	118	Irr	< 0.001	***	0.013	*	< 0.001	***	0.080	ns	< 0.001	***	< 0.001	***	< 0.001	***	< 0.001	***	< 0.001	***
		CL × Irr	0.0027	**	< 0.001	***	< 0.001	***	< 0.001	***	< 0.001	***	< 0.001	***	< 0.001	***	< 0.001	***	0.0020	**
		CL	< 0.001	***	0.21	ns	0.85	ns	< 0.001	***	< 0.001	***	< 0.001	***	< 0.001	***	< 0.001	***	< 0.001	***
	126	Irr	< 0.001	***	< 0.001	***	< 0.001	***	0.51	ns	0.37	ns	< 0.001	***	< 0.001	***	< 0.001	***	0.31	ns
		CL × Irr	0.0026	**	< 0.001	***	0.015	*	< 0.001	***	0.36	ns	< 0.001	***	< 0.001	***	< 0.001	***	0.0056	*
		CL	0.037	*	0.0016	**	< 0.001	***	< 0.001	***	0.37	ns	< 0.001	***	0.81	ns	< 0.001	***	0.015	*
	128	Irr	0.0032	**	0.70	ns	< 0.001	***	0.038	*	< 0.001	***	0.0073	**	< 0.001	***	< 0.001	***	< 0.001	***
		CL × Irr	0.0015	**	< 0.001	***	< 0.001	***	< 0.001	***	< 0.001	***	0.39	ns	< 0.001	***	< 0.001	***	0.67	ns

[a]Values are from linear mixed models with crop load (normal and low) and irrigation (deficit and full) treatments as fixed effects and block, vine and bunch as random effects. A520, absorbance at 520 nm; TSS, total soluble solids; FW, berry fresh weight; IBMP, 3-isobutyl-2-methoxypyrazine; MCPT, methyl cellulose precipitable tannin; CIRWG, colour index for red wine grapes; dpf, days-post-flowering.
